# A Review of Intelligent Fault Diagnosis for High-Speed Trains: Qualitative Approaches

**DOI:** 10.3390/e23010001

**Published:** 2020-12-22

**Authors:** Chao Cheng, Jiuhe Wang, Hongtian Chen, Zhiwen Chen, Hao Luo, Pu Xie

**Affiliations:** 1 School of Computer Science and Engineering, Changchun University of Technology, Changchun 130012, China; chengx415@163.com (C.C.); lmg0719@163.com (J.W.); 2 CRRC Changchun Railway Vehicles Co., Ltd., Changchun 130062, China; xiepu@cccar.com.cn; 3 Department of Automation, Tsinghua University, Beijing 100084, China; 4 Department of Chemical and Materials Engineering, University of Alberta, Edmonton, AB T6G 1H9, Canada; 5 Key Laboratory of Energy Saving Control and Safety Monitoring for Rail Transportation of Hunan Provincial, School of Information Science and Engineering, Central South University, Changsha 410083, China; zhiwen.chen@csu.edu.cn; 6 Academy of Astronautics, Harbin Institute of Technology, Harbin 150001, China; hao.luo@hit.edu.cn

**Keywords:** intelligent fault diagnosis (IFD) technique, qualitative approaches, artificial intelligence, high-speed trains

## Abstract

For ensuring the safety and reliability of high-speed trains, fault diagnosis (FD) technique plays an important role. Benefiting from the rapid developments of artificial intelligence, intelligent FD (IFD) strategies have obtained much attention in the field of academics and applications, where the qualitative approach is an important branch. Therefore, this survey will present a comprehensive review of these qualitative approaches from both theoretical and practical aspects. The primary task of this paper is to review the current development of these qualitative IFD techniques and then to present some of the latest results. Another major focus of our research is to introduce the background of high-speed trains, like the composition of the core subsystems, system structure, etc., based on which it becomes convenient for researchers to extract the diagnostic knowledge of high-speed trains, where the purpose is to understand how to use these types of knowledge. By reasonable utilization of the knowledge, it is hopeful to address various challenges caused by the coupling among subsystems of high-speed trains. Furthermore, future research trends for qualitative IFD approaches are also presented.

## 1. Introduction

Since the Japanese Shinkansen was born in 1964, high-speed trains had made rapid progress all over the world [1,2,3]. Due to their various advantages, such as large capacity and low energy consumption, high-speed trains have become one of the efficient tools in the transportation field, which are responsible for carrying goods and passengers [4,5,6]. Thanks to these superiorities, the most representative high-speed train techniques, like Shinkansen-N700 and E5 in Japan, China Railway Highspeed (CRH) in China, InterCityExpress (ICE) in Germany, and Train à Grande Vitesse (TGV) in France, play an essential role in the transportation of various countries [7,8,9].

A major topic of high-speed trains is its safety. In order to protect passengers to the greatest extent, a common manner is to establish reasonable maintenance strategies [10]. Therefore, issues, like fault diagnosis (FD), attract enhanced attention from academia and application aspects in the transportation field [11,12,13]. In the FD tasks of high-speed trains, there are three basic requirements [14]: judging there is a fault in high-speed trains or not, finding the original cause of faults, and forecasting fault-evolution trends. Furthermore, these achievements also provide engineers valuable references in formulating maintenance strategies [15].

However, traditional FD techniques make it difficult to diagnose some faults in high-speed trains [16,17]. One of the reasons behind this challenge is that the inherent characteristics of faults in high-speed trains could be often confused [18]. There are many kinds of faults among high-speed trains, differing in both fault symptoms and fault causes. A few faults can be directly determined by simple logic judgements [8]. Unfortunately, the features of other faults are complicated, especially for the large-scale system composed of many subsystems [19]. Moreover, when a one-one mapping relationship between fault types and their symptoms is not achieved, it will pose difficulties in successfully diagnosing faults. In fact, there are several aspects resulting in difficulties of successful detection and diagnosis of faults, which should be taken into account [20]. To sum up, these factors are listed as follows.

- Gradation. The structure of high-speed trains has several levels, including train level, system level, subsystem level, and component level. Thus, referring to gradational structure of trains, their faults and symptoms have similar features [21].
- Confusion. Aiming at complex system with the high structure coupling, the relationship among different fault characteristics is complicated. When fault occurs, factors, like redundancy and relevance, reflecting on these characteristics should be considered [22].
- Propagation. When a fault appears in systems, it is a high probability with the phenomenon of causing other systems or subsystems to fail at the same time [23].
- Uncertainty. The occurrence of faults in high-speed trains is often random. Moreover, there are still several uncertainties under the following situations, such as monitoring process of measurement data, transformation in external operation environment, and so on [24].

Apart from the above characteristics, the real-time ability of FD schemes should be taken into account. Because of these demanding requirements, traditional FD techniques make it difficult to achieve our desired results [25,26]. Until the 1980s, benefiting from developments of artificial intelligence (AI), especially for the utilization of expert system (ES), intelligent FD (IFD) techniques, regarded as a new research topic, could perform the FD tasks accurately, autonomously, and quickly [27,28,29,30,31]. Following the trend, IFD techniques have also been introduced into the transportation domain. The beginning of implementing IFD techniques needs to extract a large amount of diagnostic knowledge [32]. Fortunately, the primary requirement can be met in the FD tasks of high-speed trains due to rich knowledge that has been formed in the train maintenance and monitoring process. In comparison with the principle of traditional FD techniques (i.e., signal detection and feature extraction), IFD techniques can make use of knowledge acquisition and reasoning to obtain the FD results [33].

Many researchers have summarized the IFD approaches applied in high-speed trains on the basis of different perspectives for nearly half a century. In the early research, Frank [34] divided IFD techniques into analytical model-based, signal processing-based, and knowledge-based approaches. On this basis, Reference [35] summarized IFD approaches again according to recently emerging techniques, i.e., qualitative and quantitative approaches. Under the new classified way, both model-based and data-driven approaches are the branches of the quantitative approach. In addition, graph theory, expert system, and qualitative simulation are the branch of the qualitative approach. In particular, it is a remarkable work that IFD techniques are firstly comprehensively summarized. To clearly grasp the progress of IFD techniques in various fields, this paper provides a detailed report about the IFD works over the past 30 years, as shown in Table 1. It is remarkable that each of the researches in Table 1 has a specific diagnostic object, and the number of the approaches in every category reflects their applied situations. Unfortunately, due to Table 1 focuses on their application in various domains, instead of their classifications, the relation among these approaches is not considered.

In comparison with their applications in other areas, quantitative approaches seem not to be widely utilized in the transportation domain, mostly focusing on machine learning, multivariate statistical analysis, and signal analysis. It is well-known that three techniques mentioned above are the important branch of the data-driven method [76]. Model-based approaches, belonging to quantitative techniques, can provide effective results for trains under the accurate mathematical model [7]. However, for high-speed trains with more than 84 mathematical equations [1], its application will be limited. Without obtaining the precise input-output relationship of trains, signal analysis technique is a convenient scheme to analyze signals carrying important fault information [77]. In particular, fault symptoms are time domain, frequency domain, and joint time-frequency domain [9]. However, many signal analysis methods are most useful for the so-called steady-state systems. For the high-speed trains which switch frequently, this requirement is difficult to meet [44]. In contrast to other quantitative techniques, machine learning (ML) has recently been applied in the FD tasks of high-speed trains because of its excellent performance in processing a large amount of measurements [78]. Nevertheless, various uncertainties in practice are not considered, as well as integrated, into ML-based diagnostic frameworks. Thus, for the high-speed trains with the influence of many uncertainties, ML-based methods may not achieve the desired effect [79].

On the other hand, since they embody human intelligence, qualitative approaches are drawing considerably increasing attention in the FD domains today [80]. Following the successful implementation of the ES frameworks, qualitative techniques have been successfully applied in high-speed trains, as shown in Table 1. Roughly speaking, qualitative approaches provide a way to obtain objective and accurate conclusions by reasoning. Their implementation can be achieved without using the mathematical model or a large amount of measurement data, allowing engineers to select the most suitable qualitative techniques based on their actual needs [21]. It is enough to prove that the qualitative approach is an immense potential technique in the IFD domain. However, there is no available survey providing researchers the development and classification of qualitative techniques for high-speed trains. Therefore, it should be given a thorough examination of the development and expectation of qualitative approaches. For this purpose, four contributions in the paper are summarized as follows:

(1) The first aim of this paper is to deliver a report about the detailed structure of high-speed trains (including their common fault types and core systems) and then to provide researcher with the diagnostic objective suitable for the FD tasks of high-speed trains.
(2) The second focus of this paper is to detail the extraction path of diagnostic knowledge, where extraction rules are significant for building the high-performing knowledge base (KB). It is preparation work for using qualitative IFD methods to achieve FD proposes for high-speed trains.
(3) The third attempt of this paper is, by reviewing the research work of qualitative techniques and surveying the emerging techniques, to summarize qualitative IFD techniques from new perspectives.
(4) The final attention of this paper is, by enumerating the limitations and future investigations of qualitative IFD techniques, to inspire new research ideas for researchers and engineers.

The remainder of this paper is organized as follows. Section 2 briefly introduces the background of high-speed trains. In Section 3, a new classification of qualitative IFD techniques and their application to high-speed trains are reviewed elaborately. The limitations and future investigation of these methods are speculated in Section 4. Finally, Section 5 is dedicated to the conclusion of this paper.

## 2. Background

In this section, the core system of high-speed trains is described, and its functional component used for the FD task is listed. In addition, the acquisition path is presented because the diagnostic knowledge plays a vital role in qualitative IFD approaches. At the end, the necessary implementation steps and advantages of the qualitative IFD technique are summarized. Notice that another purpose of this section is that encouraging some researchers interested in the transportation fields is to generate new ideas to address various FD issues in high-speed trains.

### 2.1. High-Speed Trains

As a critical step in the maintenance strategies for trains, qualitative IFD techniques can ensure the safety of high-speed trains throughout the whole operation [81]. However, some IFD results are not satisfactory [82]. A major reason is that the core system of high-speed trains usually consists of multiple subsystems, where each subsystem contains the independent physical process, and the interaction between subsystems is realized by system integration [83]. It means the uncertainties from these relationships result in a complex failure mechanism, and there may be the simultaneous existence of multiple failure modes [84]. When there are unclear structures of high-speed trains, it is hard to analyze the influence of more uncertainties, such as the relationship between the structure of high-speed trains and fault propagation [29].

In fact, faults originated from different systems of high-speed trains should be classified according to the fault location. In the bogie system, there are 6 main fault modes that affect the operation of the bogie system, including abnormal axle temperature, wheel tread scuffing or peeling, mechanical stuck clamp, and so on [85]. Four subsystems are prone to faults, including primary suspension and axle box, wheel axle, secondary suspension, and bogie related inspection. In general, fault causes, together with fault modes, are various because of the different systems. For example, the traction system mainly includes the main body of the traction converter, cooling, and control of the traction converter, as well as the traction motor and its cooling. There are 12 fault modes and nearly 40 fault causes, where some errors measured by sensors have not been taken into account [86]. In other words, it is conducive to extract diagnostic knowledge for high-speed trains by clarifying their structures and fault causes. Furthermore, building a complete KB is helpful to improve the efficiency of qualitative IFD techniques.

**Figure 1:**
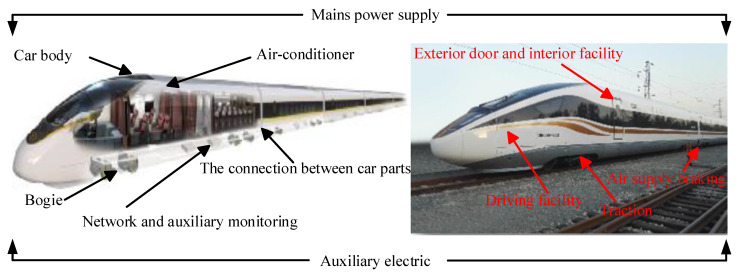
The structure distribution of important systems in high-speed trains.

**Figure 2:**
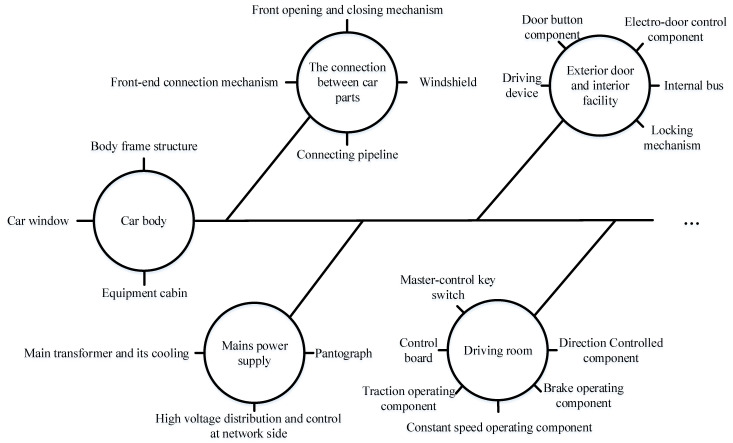
Diagram for key systems suitable as the diagnostic objective.

**Figure 3:**
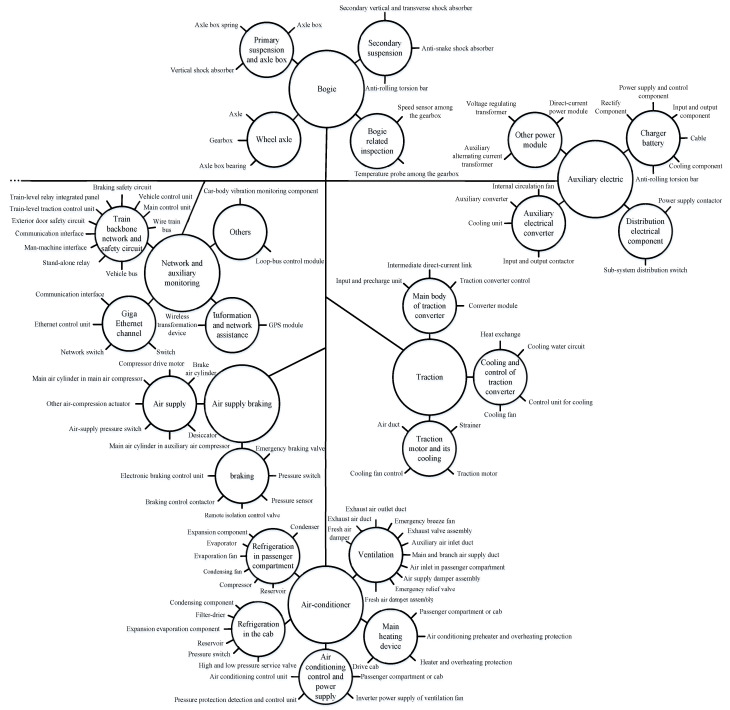
Diagram for key systems suitable as the diagnostic objective (supplement to Figure 2).

To raise researchers’ awareness of high-speed trains, Figure 1 shows a distributed system architecture of trains. The systems mentioned above can be selected as diagnostic objectives according to the research interest of practitioners and researchers. In Figure 2 and Figure 3, the core systems in the high-speed trains can be divided into multiple subsystems and components. To an end, another aim of this subsection will be the focuses on the functions of every system.

**Car body:** it is the main place for passengers and drivers to stay. Another use is the basic system for installing and connecting other equipment.

**The connection between car parts:** its core function is to connect various types of equipment which can usually affect the normal operation of trains, such as electrical or air pipeline connection between multi-section carriages, the connection between control systems and other systems, etc.

**Exterior door and interior facility:** the door is divided into the outer door and inner door according to the function and installation position. Specifically, outer doors are further divided into sliding plug doors and built-in side sliding doors. In addition, inner doors are the access doors connecting the carriages.

**Driving room:** it is the place for the drivers to control the high-speed train. According to its function, drivers can obtain the train operation information, send operation signals, and achieve various operations at the driver’s cab.

**Bogie:** it is generally divided into power bogie and non-power bogie. In reality, the bogie system is also known as a running gear system. Besides this, it is useful to pull and guide the train along the track through the electro-pneumatic braking system. Please note that it is a core system to ensure the quality and safety of train operations.

**Mains power supply:** it is the electrical equipment used in main circuits, the so-called high-voltage equipment, mainly including pantograph, vacuum break, high-voltage disconnector, and etc., transmitting the electric energy among the catenary to the electrical system.

**Traction:** it is further divided into the direct-current (DC) drive system and the alternating current (AC) drive system. Because the subsystem responsible for the control function is mainly the traction motor in traction system, it is convenient for high-speed trains to realize traction and braking functions by adjusting the torque and speed of the motors.

**Auxiliary electric:** it is generally composed of auxiliary winding of traction transformer, auxiliary converter, power socket, storage battery, and charger. Its function is to provide power for point-type equipment except for the traction power system of high-speed trains.

**Air supply braking:** its function is realized by the hybrid braking which is composed of power braking and air braking. Air supply braking system is necessary to ensure the safe operation of trains.

**Network and auxiliary monitoring:** it is generally composed of input-output network equipment and each subsystem controller and then to achieve the data transmission, sharing information, real-time control, and FD task through train control network.

**Air-conditioner:** it is generally composed of the ventilation system, refrigeration system, heating system, and operation control system. Its principal purpose is to regulate the temperature and humidity in the car through automatic control. Benefiting from its strong function, air-conditioner system can filter the circulating air, providing and maintaining a desirable internal atmospheric environment for passengers without the negative influence caused by external conditions.

However, not all systems or components are suitable for the FD tasks of high-speed trains. For instance, low electrical voltage apparatuses, like the transformer and contractor, are embedded in many core systems of trains, playing a pivotal role in some control systems. However, these types of equipment have a long life-cycle. The efficient maintenance strategy is to replace them with new components due to their low cost and large number [87]. Therefore, the significance of remaining useful life (RUL) prediction is far higher than the IFD technique, so some types of equipment, like low electrical voltage apparatuses, are not considered in this paper.

After determining the diagnostic objective, it is also important to choose efficient FD techniques. One of the basic principles is that the selected scheme could cope with the various problems of high-speed trains (e.g., fault causes are numerous and easily confused, making their sources difficult to be traced). It is worth noting that there are nearly 200 types of fault causes, involving in fatigue, corrosion, electrify malfunction, sand dust, heat, salinity, radiation, rainwater, impact, and so on. Currently, benefiting from the advantages of qualitative IFD technique, they are widely accepted by scholars as solutions in IFD tasks [88]. However, the extraction and expression of diagnostic knowledge are a difficulty for qualitative IFD techniques. The next subsection will discuss the knowledge types and extraction paths for high-speed trains.

### 2.2. The Classification and Expression about Diagnostic Knowledge in High-Speed Trains

The effective knowledge extraction and conversion are the basis on which qualitative IFD techniques are built. To our knowledge, diagnostic knowledge of high-speed trains is knowledgeable and has characteristics of diversity, complexity, and non-standard. To some extent, these features increase the difficulty in using the diagnostic knowledge (in terms of knowledge induction, knowledge sort, knowledge mining, and knowledge application) but reducing the knowledge reusability and integrability [23]. In other words, the quality of knowledge acquisition and the scale of KB construction are still the challenges in the application of qualitative IFD techniques to high-speed trains. To extract high-quality knowledge from the structure and mechanism from high-speed trains and establish a complete KB, the process of knowledge acquisition should comply with the following conditions:

- Easy to be understood and implemented. Each means of expression should be consistent with the human logical thinking so that it can be easily described by the computer.
- Easy to use knowledge for reasoning. The objective of knowledge expression and knowledge storage is used for knowledge for reasoning, and further analyzing the fault causes. If the structure of knowledge expression is complex or the knowledge is difficult to understand, it will reduce the diagnostic efficiency.
- Beneficial to the management of KB. During the operation of the system, some knowledge will be increased or reduced according to the actual situation. Therefore, the selection of appropriate knowledge expressions could reduce the difficulty of knowledge management and improve the efficiency of knowledge maintenance.

These are the most basic conditions about knowledge expression using for qualitative IFD techniques, and diagnostic knowledge in high-speed trains can be acquired in the following ways [89]: (1) Artificial knowledge. As a common knowledge in transportation domains, it should be obtained by experts, to combine the causal relationship between faults and symptoms. Furthermore, it is convenient to store the diagnostic knowledge into the KB by converting knowledge into rules. Finally, with the help of domain experts, these types of knowledge is progressively refined until the desired KB is established; (2) Semi-automatic knowledge. As a knowledge requiring to be standardized by experts, it should be imported from the domain experts to the KB through the knowledge acquisition system; (3) Automatic knowledge. As a knowledge requiring to be acquired by a self-learning system, it should be extracted from the original information provided by domain experts. Furthermore, the experience is summarized from the actual operation of high-speed trains to obtain new diagnostic knowledge. On this basis, these procedures contribute to the completeness of the original KB.

**Figure 4:**
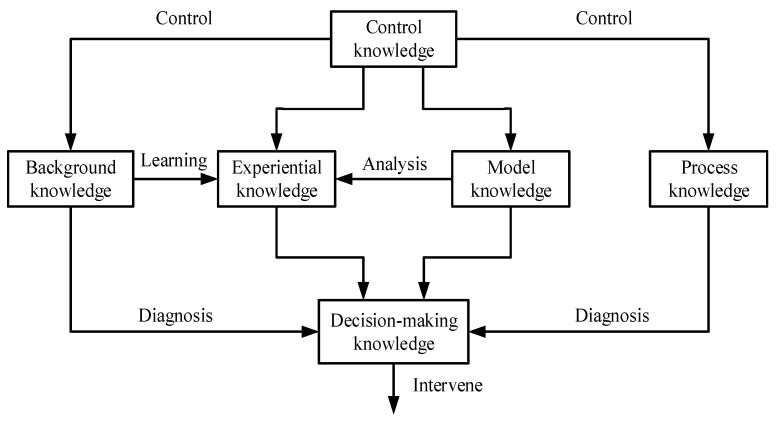
The relationship among various types of diagnostic knowledge on high-speed trains.

In addition, the realization of intelligent reasoning using qualitative IFD techniques is also based on the premise of successfully acquiring domain knowledge. In general, diagnostic knowledge is the experience summary accumulated in the long-term practice of production and life in practice. Apart from the extraction path, learning and mastering the relationship among various types of diagnostic knowledge can be helpful to enhance the quality of the KB. Thus, the purpose of Figure 4 is to strengthen researchers’ understanding of diagnostic knowledge by drawing the logical relation diagram relationship among various types of knowledge [23]. To be specific, diagnostic knowledge in high-speed trains is mainly converted into rule and case sets which can represent causality between symptoms and causes, since such knowledge expressed in this way facilitates the implementation of qualitative methods. On the other hand, system structure can also be seen as a kind of generalized knowledge which can be used flexibly by researchers. For example, in Reference [90], a qualitative IFD method based on fault tree is established according to system structure diagrams. Here, fault tree is a typical qualitative diagnostic KB [58].

**Figure 5:**
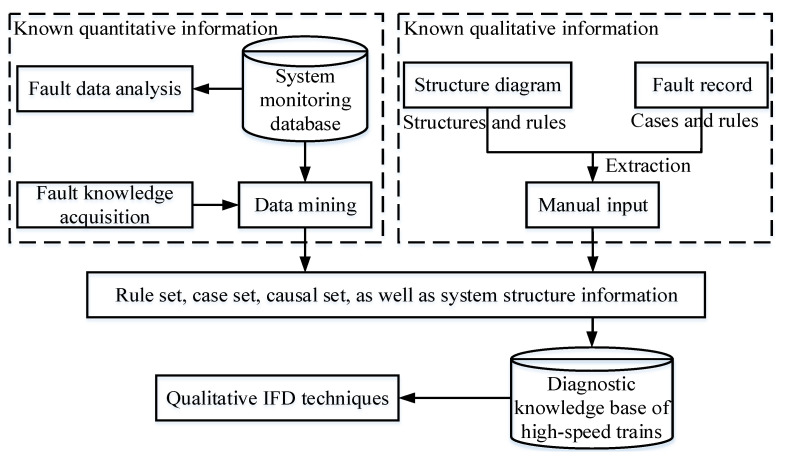
The construction of diagnostic knowledge from high-speed trains.

**Figure 6:**
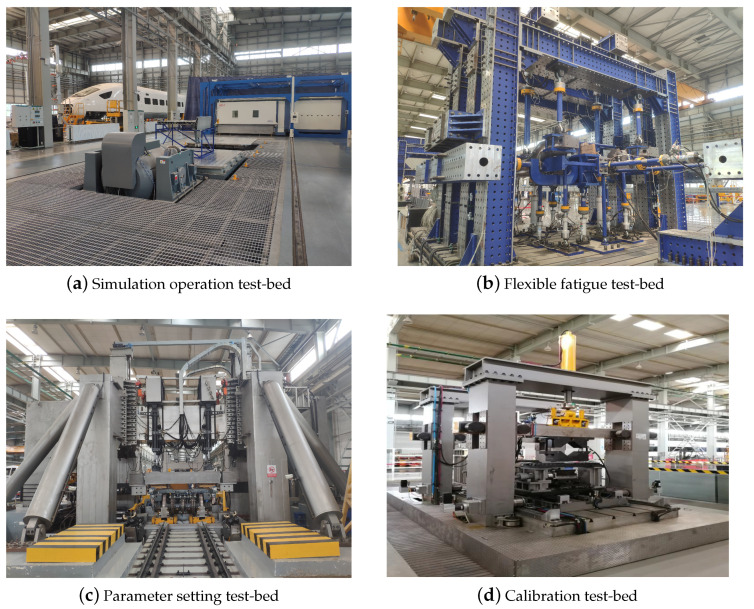
Experimental platform of high-speed trains.

As one of the key steps to build a diagnostic KB, fault knowledge extraction should be carried out from two aspects, i.e., known quantitative and qualitative information in high-speed trains, as shown in Figure 5: (a) known qualitative information mainly refers to maintenance manuals, fault records, characteristic parameter manuals, electrical schematic diagrams, system structure diagrams, etc. They are derived from the historical maintenance records and mechanisms of high-speed trains. The above information is intuitive and easily translated into usable diagnostic knowledge through manual inputs. In comparison with known quantitative information, qualitative IFD techniques seem to prefer rule and case sets extracted from known qualitative information because this kind of knowledge is more suitable for reasoning mechanisms. The function of causal set extraction is to open up the interpretable knowledge network, and another benefit of causal sets is to provide knowledge for qualitative causal IFD techniques designed for specific systems; (b) known quantitative information is mainly the monitoring dataset collected from the onboard information system in the train operation or the simulation operation test-bed in the lab, as shown in Figure 6. A large amount of potential useful fault knowledge hides in the fault dataset. However, these data cannot be used directly because fault data points can only be fully utilized by engineers after they are converted into useful knowledge. Thus, data mining algorithms are used to extract fault knowledge from a dataset and stored in diagnostic KB. The typical mining methods used for extracting knowledge are mainly divided into the following categories:

(1) Decision tree (DT): it is a classification model developed in machine learning, and its principle is that the classification rules in the form of DT are deduced from a set of unordered tuples. The classification model of DT is a directed acyclic tree, where a greedy algorithm is a basic approach to construct DT, mainly adopting top-down recursive form. In Reference [91], an unsupervised fault tree is developed to extract knowledge from data and form fuzzy rules. With the help of DTs, Reference [92] constructs a database containing a lot of experimental data.

(2) Clustering: it can divide the monitoring data into several different clusters according to the different characteristics of data. After this division, the differences among elements in the same cluster are small; elements in different clusters can vary greatly. Reference [93] uses a clustering method to help engineers discover fault states in historical data, to collect useful knowledge, as well as build a diagnostic KB.

(3) Artificial neural network (ANN): it can simulate the human brain to retain information. ANN consists of a set of input/output units connected by weights. Potential rules and connections between data are discovered and learned by adjusting the weights. Reference [94] presents an ANN methodology for fuzzy rule extraction by following the line of rule extraction investigation.

(4) Rough sets theory (RST): it is a tool for studying inaccurate and uncertain knowledge. RST expresses imprecise knowledge through the concepts of upper and lower approximation, mainly used to discover the internal relationship of inaccurate data. For example, Reference [95] presents a RST method to extract rule sets from data, where the size of decision tables is reduced through rule-joining operations. In Reference [96], a vague decision information system (VDIS) is constructed based on a vague-rough set method, and this model can extract decision rules from VDISs.

(5) Genetic algorithm (GA): it is a search method inspired by biological evolution, where GA combines the “survival of the fittest” principle with the random information exchange mechanism of chromosomes within the group. Through iterative optimization, GA can search and mine enough useful information from data. Reference [97] proposes a hierarchical genetic algorithm to extract rule-based knowledge from the observations. Combined with GA, Reference [98] develops a two-phase hybrid evolutionary classification method to extract classification rules.

(6) Support vector machine (SVM): it is a learning approach based on the principle of structural risk minimization. The main problem of SVM is to find an inductive principle that minimizes risk. Compared with the traditional methods (like many machine learning approaches based on experiential minimization), SVM has a stronger generalization ability. Reference [99] presents the fuzzy rule extraction strategy. According to several divided controllable domains, the fuzzy control rules from different domains are extracted by using SVM. In addition, the authors in Reference [100] propose a rule extraction method based on SVM by analyzing the distribution of samples.

(7) Fuzzy sets theory: different from the classical set, it has no precise boundaries. Its membership function can map the elements in the fuzzy set into 1 or 0. When a membership is equal to 1 or 0, this set is the classical set. Thanks to this thinking, fuzzy sets, regarded as a data mining method, can be used to extract knowledge and form fuzzy rules. Based on the powerful fuzzy theory, Reference [101] develops fuzzy classification systems to extract fuzzy rules using the formal concept analysis theory.

To summarize, any kind of known information collected from high-speed trains needs to be transformed into rule, case, or causal sets as useful knowledge. When a diagnostic KB is successfully constructed, qualitative IFD techniques can provide the desired results with the help of knowledge.

### 2.3. Advantages of Qualitative IFD Methods

**Figure 7:**
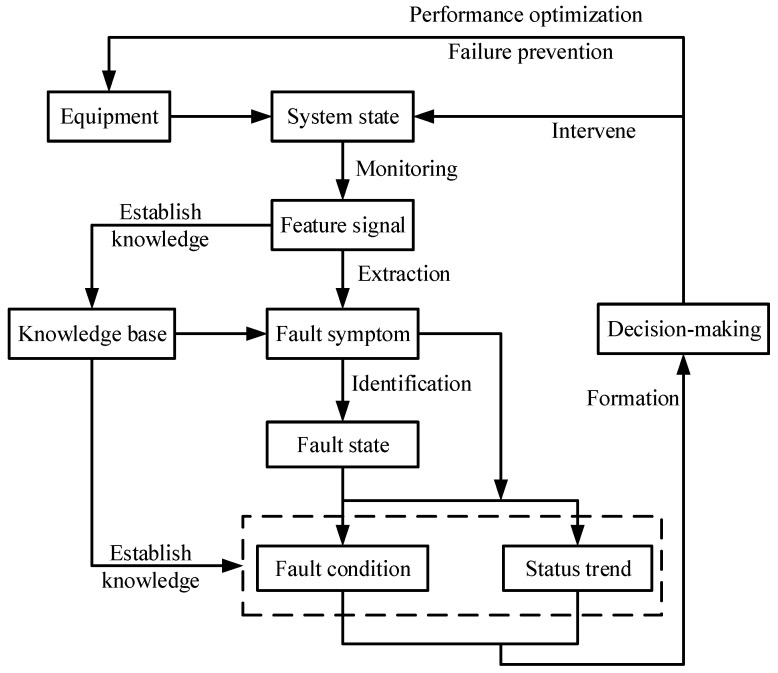
A schematic description of qualitative intelligent fault diagnosis (IFD) modeling.

The purpose of using qualitative IFD techniques in high-speed trains is, by determining the causes and locations of the fault appearing in trains, to take effective measures which can ensure the safe train operation. Some faults appearing in high-speed trains can be detected by analyzing fault symptoms during the train operation. Furthermore, with the help of the reasoning strategies and expert experience, the most effective way to improve the diagnostic accuracy is achieved by determining and excluding potential faults one by one. The application of qualitative IFD techniques should run through the whole life-cycle of the equipment on high-speed trains (i.e., from train design to maintenance). Therefore, the familiarity with the whole IFD modeling process contributes to its implementation to high-speed trains, as shown in Figure 7. In comparison with other techniques, the qualitative IFD technique has some advantages as follows [102]:

- It is easy to be designed and used.
- It is especially suitable for FD tasks with sufficient data.
- It can be implemented without accurate parameters.
- It can provide the basis for the diagnostic results (because a strong causal relationship exists among various fault modes and fault causes).
- It can make clear and transparent reasoning in uncertain situations.

Qualitative IFD techniques are close to the logical thinking of human beings, by integrating the knowledge and experience of experts into the FD tasks, to diagnose and detect faults in high-speed trains. Benefiting from the development of artificial intelligence and computer techniques, applications of qualitative IFD approaches have become more and more popular.

## 3. Applications of Qualitative IFD Approach in High-Speed Trains

Previous work, such as Reference [58], has divided qualitative IFD techniques into three categories: expert system, fault tree, and directed graph. However, it is essential to provide a new classification and summarization of qualitative IFD techniques, particularly in the emergence of advanced techniques, to keep abreast of advances in qualitative IFD approaches, as well as to form the new FD schemes to address various traffic engineering challenges.

**Figure 8:**
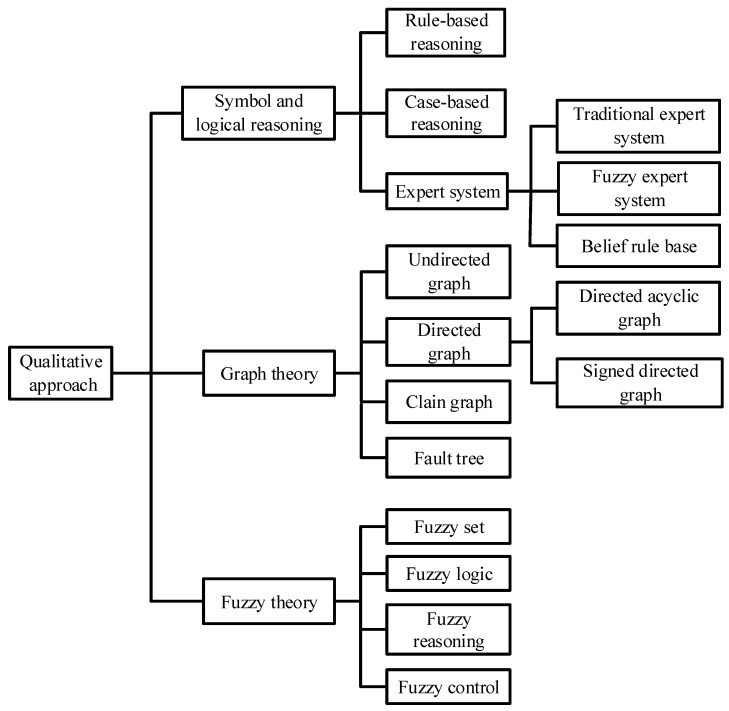
A new classification of qualitative IFD technique mainly applied in high-speed trains.

On the basis of the existing survey papers, a new classification of qualitative IFD approaches can be given in this section and is shown in Figure 8. Note that the research progress of qualitative IFD techniques can be described mainly from two aspects (i.e., theoretical background and application status in high-speed trains).

### 3.1. Symbol and Logical Reasoning in Qualitative IFD Approach of High-Speed Trains

Thanks to the primary advantages of reasoning techniques (i.e., consolidating and developing new knowledge from existing knowledge), symbol and logical reasoning approaches are gaining popularity in IFD applications to high-speed trains [103]. To start summarizing these reasoning methods from the theoretical and practical viewpoints, a quality modeling of high-speed trains is necessary, as depicted in the following subsections.

#### 3.1.1. Rule-Based Reasoning

As a general reasoning approach, rule-based reasoning (RBR) is achieved by transforming the experience from domain experts into rules, as well as using heuristic knowledge to reasoning. It is the necessary precondition for practitioners and researchers to grasp the basic knowledge of the transportation field [104]. Similar to other qualitative IFD methods, RBR approach can excavate the inner link between phenomenons and causes of faults by combining relevant knowledge, in which advantages are the natural expression and good interpretability. In the high-speed trains, faults and the observable symptoms usually follow a physical causality. Naturally, these relationships can be quantified [105].

However, some basic physical relations are neither obtained by analysis, nor by complex calculations. In general, diagnostic knowledge should be expressed in the form of rules, in which the most classical rule is the IF-THEN rule, as follows:

(1) $$\begin{array}{c} IF<condition>THEN<conclusion>, \end{array}$$

which establishes a KB.

Here, it is remarkable that: (i) the condition, also named as the premise, contains facts in the form of observed symptoms as inputs; and (ii) the conclusion, including two possibilities (i.e., events and errors), describes the logical cause of events. Rules, linked by logic “∨” or “∧”, could be independent of each other. Furthermore, these rules have a hierarchical relationship in the KB. It is worth mentioning that RBR approaches center on rule transformation and reasoning, through the effective utilization of the transportation domain knowledge, to achieve the FD tasks of high-speed trains.

With the development of traditional IF-THEN rules, other methods based on reasoning approach with IF-THEN rules have been developed gradually. Reference [106] proposes a new emerging concept, the so-called distributed fuzzy IF-THEN rule, and in which all possible rule expressions can be formed according to the different fuzzy partitions. To illustrate the effectiveness of RBR in the real-time FD tasks, a new reasoning approach with IF-THEN rules is developed and implemented by Reference [107], successfully applied in a real-time FD platform. Moreover, a causal chain used in the reasoning method can further enhance diagnostic performance.

Although the reasoning approaches with the traditional IF-THEN rules have achieved the satisfactory results, they are not interested in the external relationship among state variables, and in the internal relationship among various types of information. Obviously, when a fault appears in high-speed trains, there may be multiple variables affected. In most cases, other state variables are also implicated [108]. Therefore, it is necessary to make a comprehensive analysis of these state variables. According to the obtained results, the operation status and potential faults of high-speed trains are further judged. For this purpose, reasoning approaches with ARs have also attached more attention.

Association rule (AR) is a method to discover the relationship among itemsets in massive amount of measurement data [109]. In the FD tasks of high-speed trains, itemsets can be described as symptom sets, fault sets, and so on. To some extent, after extracting the rules from the diagnostic knowledge, the reasoning approaches with ARs can predict future behaviors by discovering the interrelated rules. One obvious advantage of ARs is that fault causes or influence factors of the core systems can be found out, with the help of the so-called association relationship. Indeed, the analysis of historical faults and state variables is an essential process in most cases, and the analysis results can be used in the maintenance plan of high-speed trains.

Suppose a set contains valuable faults and state variables. The basic form of the set is a Boolean number, indicating whether there is a fault in trains. In this way, each fault can be represented by a Boolean vector. Some knowledge reflecting the frequent itemsets of these types of information can be obtained by analyzing the Boolean vector. On the basis, these types of knowledge can be described as ARs. The rule support and confidence are two interest measures of rules [110]. Taking fault A⇒fault B as an example, A⊂T, B⊂T, and A∩B=∅. *T* represents the transaction items in transaction database *D*, and the number of all transaction items *T* in the database is |D|. The probability of itemsets in *T* is p(A). The support degree of fault A⇒fault B is the proportion of A∪B in *D*, which can be expressed by the probability:

(2) $$\begin{array}{c} Supp\left(A\Rightarrow B\right)=P\left(A\cup B\right)=\frac{p(A\cup B)}{\left|D\right|}. \end{array}$$

The confidence degree of fault A⇒fault B is the ratio of both *A* and *B* in *D*, which can be expressed by the conditional probability:

(3) $$\begin{array}{c} Conf\left(A\Rightarrow B\right)=P\left(B|A\right)\frac{p(A\cup B)}{A}. \end{array}$$

Next, the concepts of minimum support and and minimum confidence are further defined as $S{\left(A\Rightarrow B\right)}_{min}$ and $C{\left(A\Rightarrow B\right)}_{min}$, respectively. In addition, interest degree is based on the ratio of real strength to expected strength under the assumption of statistical independence:

(4) $$\begin{array}{c} I=\frac{S(AB)}{S(A)S(B)}, \end{array}$$

where I≥0, and when *I* is far greater than 1, which means that this rule is of more importance.

With the development of data mining techniques, different types of AR are gradually proposed. One of ARs is Apriori algorithm [111]. Benefiting from the development of Apriori algorithms, reasoning approaches with ARs have been found useful in designing FD schemes for high-speed trains. With the help of the cloud computing platform, Reference [112] develops an IFD approach by combining the traditional Apriori algorithm to diagnose faults in trains. In addition, due to the coupling relationship that must be taken into account, Reference [113] designs a new IFD scheme which combines mining ARs with parallel ARs, to diagnose faults in subsystems of CRH2 trains. It is worth noting that the proposed approach in Reference [113] is a distributed IFD scheme, and reasoning approaches with traditional IF-THEN rules are difficult to detect these faults appearing in high coupling systems.

Naturally, aiming at realizing maximized fault detectability for high-speed trains, whether reasoning approaches with IF-THEN rules or reasoning methods with ARs, it is necessary to enhance the FD performance by combining AI techniques.

#### 3.1.2. Case-Based Reasoning

Different from RBR methods, case-based reasoning approaches (CBR) are ideal for inferring solutions to FD tasks that have many representative cases [114]. Roughly speaking, the CBR method is, by matching similar FD cases in a case base, to learn new knowledge, as well as perform FD tasks of high-speed trains. In other words, it is suitable for insufficient domain knowledge and inconsistent fault descriptions. In the case base of CBR, each case is the accumulation of phenomenons and causes of faults in practice. Reference [115] provides the simplified calculations of CBR: (1) case representation and index; (2) case retrieval; (3) case revision; (4) case study.

**Figure 9:**
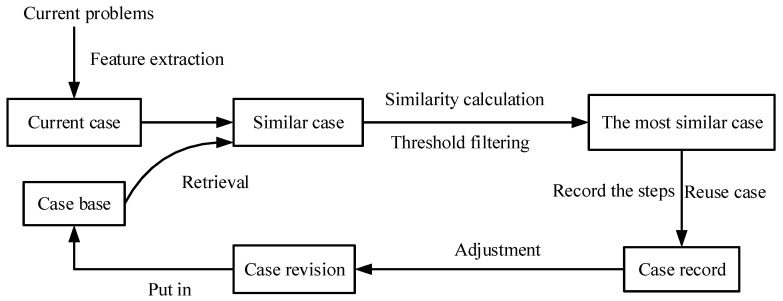
A schematic description of reasoning approaches (CBR) modeling.

In general, currently resolved faults are made into cases and stored in a case base in accordance with the standard format. When there is a new FD task, the result can be obtained by means of retrieving the case base and matching all similar cases. If the fault mode (from a new FD task) and the case fail to match, additional matching methods will be adopted to deal with this situation. Until the fault mode is successfully matched, this result is added to the case base as a new case after standardized processing. Figure 9 illustrates the key steps in designing the CBR solutions.

In addition, CBR is easy to acquire diagnostic knowledge and easy to be understood. These advantages make CBR approaches more suitable for situations that need a lot of experience, such as high-speed trains. Due to many maintenance records are recorded in train operations, more and more attention has been attached to CBR approaches used for FD tasks in high-speed trains. For instance, aiming to carry out the maintenance operation for faulty equipment, the authors in Reference [116], by annotating the fault features recorded during system maintenance, employ the CBR framework to diagnose faults in vehicle onboard equipment of high-speed trains. On this basis, Reference [117] develops a new intelligent maintenance decision system, by integrating into CBR methods, to obtain the desired FD results of CTCS-300T vehicle onboard equipment in high-speed trains.

The key of CBR methods is to construct an effective search case. For improving the coverage rate of FD cases in CBR, a feasible scheme is to make the cases cover as many solution spaces as possible and then to significantly improve the FD performance of CBR.

#### 3.1.3. Expert System

In qualitative IFD approaches based on the symbol and logical reasoning, the availability of FD results depends on knowledge and experience accumulated by engineers [118]. Unfortunately, these experiences are difficult to express in the traditional “process-oriented” or “object-oriented” programming. Consequently, there is an urgent need for a “knowledge-oriented” technique to express these types of knowledge [119].

Among many “knowledge-oriented” approaches, expert system (ES) is very suitable for expressions of diagnostic knowledge. Because it is able to leverage the advantages of experts and computers, ES can further reduce the influence of human factors in FD tasks. Benefiting from its advantages, like intuitive expressions, the unified form, strong modularity, and simple reasoning mechanism, ES applied in high-speed trains has gained attention in academia [120,121,122].

**Figure 10:**
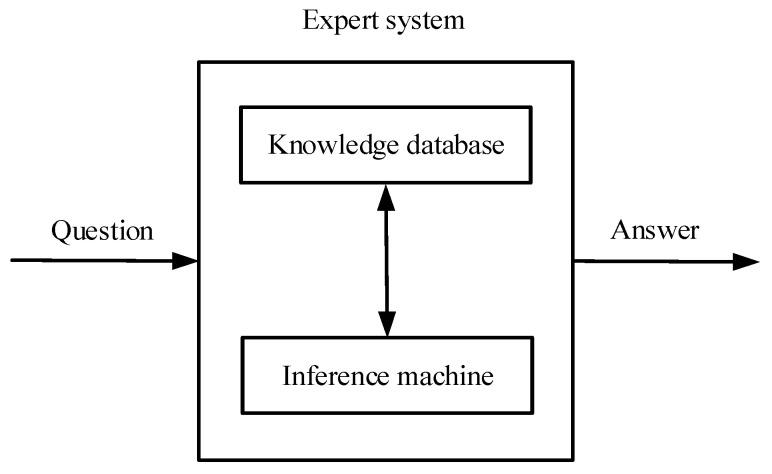
The composition of simplified expert systems (ESs).

Combined with knowledge-based programming approaches, ESs usually have an immense KB with some diagnostic experiences and reasoning mechanisms [123]. Specifically speaking, a complete ES includes a knowledge database, inference machine, comprehensive database, man-machine interface, and interpretation module [35]. To our knowledge, another simplified form of ESs is regarded as the combination of “knowledge database” and “inference machine”, as shown in Figure 10. In general, there are two kinds of knowledge, the so-called background of rules, storing in a KB: (1) it contains the knowledge related to the faults; (2) it uses the domain knowledge to infer the FD tasks. Moreover, inference machine could match FD facts with diagnostic knowledge to deliver robust FD results [124].

Benefiting from the utilization of RBR and computer techniques, traditional ESs have been well-developed in high-speed trains, which often uses IF-THEN rules to describe domain knowledge [125]. In the early work, Reference [126] firstly introduces the traditional ES into FD fields, where faults in a boiling water reactor are successfully detected. In order to form a complete ES theoretical system, Reference [127] provides a detailed report from the following aspects: (1) hierarchical structures of knowledge expression; (2) characteristics of knowledge expression, to build the theoretical and practical basis of ESs. Afterwards, ESs are extended to transportation domains [128], where a traditional ES scheme performs the FD tasks in brake systems according to the accumulated experience and measurement data in high-speed trains.

However, all expert knowledge is not accurately expressed, mainly affected by various uncertainties. On this basis of fuzzy theory, fuzzy ES (FES) has gained vast progress [129]. By combining with fuzzy membership degree and fuzzy reasoning, FESs can deal with the uncertainties of expert knowledge with a better performance. For instance, Reference [130] uses fuzzy processing to combine accumulations and deviations of system parameters, by distinguishing false alarms from actual faults, to improve the performance of FD results. From another perspective to obtain the expected FD results, a FES scheme is developed in Reference [131], where the inference engine in this scheme is composed of Chinese character segmentation and fuzzy reasoning. In addition, due to parameters, like membership degree and credibility, considered in FES modeling, this approach can improve FD performance.

Slightly different from fuzzy theory that can deal with uncertainties caused by inaccurate knowledge, evidence theory mainly deals with uncertainties caused by unknown knowledge [104]. Based on several critical techniques, like fuzzy theory, evidence reasoning, and decision theory, the belief rule base (BRB) is proposed in Reference [132]. Afterwards, Reference [76] further develops BRB-based methods, and systematically proposes the structure optimization techniques to optimize initial parameters in BRB approaches. In essence, BRB is an ES based on If-Then rules, by combining with belief, to describe causal relationships under the influence of various uncertainties. Currently, BRB methods have many studies on FD tasks of high-speed trains. To reduce the complexity of BRB, Reference [24] develops a BRB approach, by combining with principal component analysis technology (PCA), to detect faults in running gear systems of high-speed trains. On this basis, Reference [21] designs a BRB with mixed reliability (BRB-mr) scheme, by quantifying two types of uncertainty factors in the process of sensor measurements, to diagnose faults in running gear systems.

Following knowledge used in ESs, the above ESs belong to ESs based on shallow knowledge (mainly from the experience of experts). Moreover, solutions based on shallow knowledge to FD issues generally need two kinds of knowledge, i.e., one is how to cause various symptoms when a system fails; the other reflects the level of causal relationships. In spite of some works that have extended to transportation domains, most of them can only detect system level or component level faults in high-speed trains, and train-level faults are difficult to be detected. One of the reasons is that high-speed trains have many subsystems, in which faults caused by many factors are difficult to analyze through IFD techniques based on shallow knowledge. In other words, it is necessary to extract deep knowledge of high-speed trains, such as mechanism and coupling among subsystems.

Based on the analysis above, it is clearly known that FD schemes applied in train level faults need to obtain model knowledge from high-speed trains. Furthermore, ESs based on deep knowledge are established. When actual outputs in a FD task are inconsistent with the expected outputs, a set from the above inconsistent outputs is established. According to domain knowledge and the coupling among systems, all possible sources of failures can be found through ESs based on deep knowledge.

However, ESs based on deep knowledge also have certain limitations. On the one hand, they have many characteristics, such as large-search space and slow-reasoning speed. These characteristics make the ES based on deep knowledge difficult to be applied to high-speed trains as an independent technique. On the other hand, with the applications of AI to FD domains, ESs based on the combination of deep and shallow knowledge have attracted tremendous attention [133]. ESs based on the combination of deep and shallow knowledge are the advanced IFD techniques, mainly including integrated FD expert systems, hierarchical causal models, and multi-agent ES techniques. In future research, these techniques will be one of the active topics.

### 3.2. Graph Theory in Qualitative IFD Approach of High-Speed Trains

Graph theory, making use of graphic structures to describe models, is a multivariate analysis technique that originated from the path analysis and the statistical physics [134]. To solve uncertainty and complexity problems in engineering applications, the work in Reference [135,136] developed FD approaches based on graphs through the combination of probability and graph theory.

From the topological structure, the graph approach considering casual relationships, in essence, is a logic diagram [137]. To be more precise, its nodes represent facts in FD tasks (e.g., causes, symptoms, locations of faults), and its edges are used to describe probabilistic relationships among each graph node. It is worth noting that these probabilistic relationships can be quantified (because they are usually hidden in conditional probability density functions among nodes). Thus, descriptions and reasonings of uncertainty issues can be carried out through a uniform theoretical framework [138]. Moreover, by judging whether connections among each node are undirected or directed edges, graph methods can be divided into undirected and directed graphs.

However, mixed graphs are introduced based on undirected and directed graphs, to meet requirements of graph models in high-speed trains, mainly including the undirected edge (–), the directed edge (→), and the bidirectional edge (↔) [139]. In fact, a graph model is a family of probability distributions, where each probability distribution should satisfy an independent set of conditions encoded by graphs. Suppose R is a finite set of non-empty vertices (variables), and U,V∈R (U≠V). A set of edges is $\varepsilon =\left\{--,\to ,\leftarrow ,↔\right\}$, and B(ε) represents a density set ε. A mixed graph G=(R,E) is an ordered pair of the vertex set R and mapping E:R×R→ B(ε), satisfying:

(5) $$\begin{array}{cc} \; & E(U,U)=\emptyset \\ \; & -\in E(U,V)\Leftrightarrow -\in E(V,U)\\ \; & \leftarrow \in E(U,V)\Leftrightarrow \to \in E(V,U)\\ \; & ↔\in E(U,V)\Leftrightarrow ↔\in E(V,U). \end{array}$$

Here, vertex sets and edge sets of mixed graphs can also be written as R(G) and E(G), respectively. The relationship among vertices in the graph *G* could be described as: If the graph has the edge $\left\{U-V,U↔V,U\to V,U\leftarrow V\right\}$, then *U* is $\left\{neighbor,spouse,parent,child\right\}$ of *V*, as well as named as $\left\{U\in ne_{G}\left(V\right),U\in sp_{G}\left(V\right),U\in pa_{G}\left(V\right),U\in ch_{G}\left(V\right)\right\}$. When $V^{{}^{\prime }}\subseteq V$ and $E^{{}^{\prime }}\subseteq E$ both are satisfied, G=(V,E) and $G^{{}^{\prime }}=\left(V^{{}^{\prime }},E^{{}^{\prime }}\right)$ are mixed graphs, and $G^{{}^{\prime }}$ is called a subgraph of *G*.

#### 3.2.1. Directed Graph

Directed graph (DG) techniques, composed of node variables and directed edges, can describe qualitative relationships among variables to detect faults in high-speed trains through reasoning strategies of consistent path theory.

As the most basic DG models, signed DG (SDG) is adopted in describing causalities of systems [140]. Different from other techniques, SDG can be implemented without precise matrix descriptions or complete measurement data because it uses a comprehensive graphical form in FD procedures [141]. Besides, SDGs can simply analyze fault propagation principals for high-speed trains.

Denote SDG as $\chi =\left\{G^{{}^{\prime }},\delta ,\phi \right\}$, where $G^{{}^{\prime }}$ is a DG, δ is the state function of nodes, and ϕ is the branch symbol function. Symptoms or characteristics of faults in systems are represented by nodes, in which causalities among variables are described through directed edges from cause nodes to result nodes. When a system fails, the state of fault nodes deviates from normal values. Furthermore, an alarm is triggered due to the deviation. Thus, by analyzing the causes of node changes, the possible propagation paths of faults can be found. Based on these paths and known causes of faults, the evolution process of faults in systems can be discovered.

Aiming at different problems, various SDG methods for high-speed trains have been developed in FD fields. A fundamental challenge in high-speed trains is the optimal configuration of sensors in different systems. For this reason, Reference [142] designed an IFD scheme, based on SDG, to perform two tasks for braking systems in trains, i.e., FD and optimal configurations of sensors. Besides, the extended work in Reference [143], also named as a symptom-fault association-based method, is to reduce the complexity of SDGs. With the continuous expansion of high-speed trains, the single SDG technique is difficult to deal with dynamic changes of abnormal variables in systems. Therefore, Reference [144] firstly introduced multi-layer strategies into SDGs and then developed a three-layer SDG framework to detect faults of the system state transition caused by a single cause.

In Reference [145], three salient advantages of the SDG are summarized: (1) it can deal with the closed systems; (2) it can deal with uncertainties, incomplete information, and noise; and (3) it is easy to be built. Although SDG has better FD performance in the optimal configuration of sensors and fault propagation path analysis, it is difficult to be used for analyzing faults in several high coupling units (or subsystems, or components) according to the train structure.

Another important subset of DGs, the Bayesian network (BN), is proposed on the basis of the directed acyclic graph (DAG) and the conditional probability table (CPT) [146]. Due to that fact that BNs can be used for IFD applications in highly coupling trains, they are gaining popularity.

Taking the component in trains as an example, there are various characteristic signals (e.g., pressure, speed, vibration, etc.) carrying with important information among components. Within a small area consisting of several high coupling components, the components that generate and deliver these signals can be regarded as information sources, and information exchanges among components are accomplished through non-component carriers. Once the information exchange is abnormal, it can be inferred that the component regarded as the information source is faulty. For instance, in order to control braking systems, air compressors feed the gas directly into cylinders and then adjust the pipe pressure of train pipes through an automatic control valve. If air compressors are abnormal, the pressure in cylinders will be directly affected. Explicitly, the pressure is the information source for the coupling of two components. Besides, there are no other valves or relays among them.

Similar to direct couplings, indirect couplings also exist in components, i.e., there is no direct information exchange between two components. Suppose the component $A_{1}$ can transfer information to the component $A_{3}$ through the component $A_{2}$, achieving the information exchange, where there are two direct couplings, including $A_{1}\to A_{2}$ and $A_{2}\to A_{3}$. It is consistent with the principle of directed acyclic. Thus, a DAG and a set of random variables in B=(G,θ) are needed to describe the above coupling, also named as the conditional dependency in BNs, in which *G* represents a DAG.

It is clear that qualitative descriptions of dependencies among features exist in B=(G,θ), which is composed of nodes and directed edges from the parent node to the child node [147]. Furthermore, nodes in *G* are fault characteristics in systems, and directed edges represent dependencies among fault characteristics. Note that a specific case without connections through directed edges means that faults are independent of each other. The work in Reference [148] demonstrates that BNs use graphs to encode the conditional dependency among variables in the probability distribution, also named as Markov property of graphs. Define θ as a set of conditional probabilities describing the network distribution, where θ shows dependencies among each characteristic and its parent node.

Denote a set (mainly including fault characteristics in core systems of high-speed trains) as $U=\left\{U_{1},U_{2},\ldots ,U_{n}\right\}$, where $U_{i}\left(i=1,\ldots ,n\right)$ represents each node in the network. In order to achieve FD tasks using BNs, the first step is to determine relationships between fault modes and nodes in networks. Then, the DAG that meets independent conditions is further established in (6):

(6) $$\begin{array}{c} p\left(U\right)=\prod_{i=1}^{n}p\left(U_{i}|U_{1},U_{2},\ldots ,U_{i-1}\right). \end{array}$$

**Figure 11:**
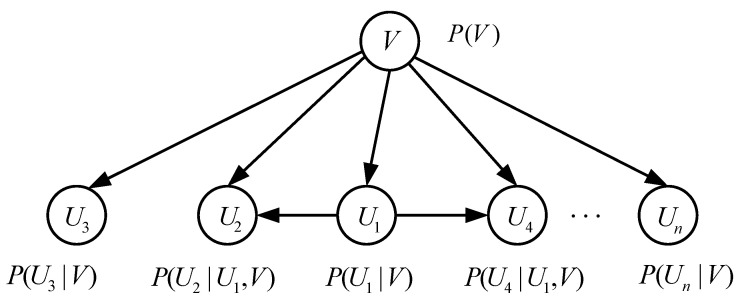
Illustrations of Bayesian network modeling.

Moreover, the joint probability distribution of *U* is given:

(7) $$\begin{array}{c} p\left(U\right)=\prod_{i=1}^{n}p\left(U_{i}|pa_{G}\left(U\right)\right), \end{array}$$

in which $pa_{G}\left(U\right)$ is a set of parent nodes in the *U*. Furthermore, the set of fault characteristics is extended to $U=\left\{U_{1},U_{2},\ldots ,U_{n},V\right\}$, and *V* is defined as a set of fault types. Figure 11 shows a simple modeling procedure of BNs.

When a fault occurs, the joint probability distribution of variables is described as the product of all variables under the variables of parent nodes, the vertex set containing faults can be uniquely identified as long as its probability of the fault exceeds the fault threshold “*t*”. The above FD procedure is also named as the *t*-probability diagnostic system [149]. The joint probability distribution in BNs has certain physical significance and is convenient for FD applications in high-speed trains.

One of the earliest applications of BNs to high-speed train is from Zhou et al. [150]. They employ BNs and fuzzy reasoning to detect faults in control systems of high-speed trains. Considering that most of the complex systems in trains have dynamic degradations, it is difficult to directly diagnose faults in a special case, the so-called multi-transition states. For this reason, Reference [151] proposes a new dynamic BN. With the help of Markov chain, the dynamic BN approach can detect transient faults and intermittent faults in control systems, providing researchers with fault symptoms of systems at a specific time. Similar to Reference [144], Reference [152] develops a multi-layer BN framework, by combining with bond graphs, to analyze fault propagations of traction systems. Different from other work, Reference [153] designs a mixed IFD approach, which is the combination of static and dynamic BNs, achieving real-time FD tasks of control systems in trains.

In BNs applied to high-speed trains, static and dynamic BNs are suitable for addressing different challenges. Specifically, static BNs are useful to solve uncertainty issues in fault location. On the contrary, dynamic BNs are often used for train maintenance.

#### 3.2.2. Undirected Graph

Undirected graph (UG) approaches, also known as Markov networks, are mainly originated from statistical physics. In UGs, undirected edges are usually used to describe the direct probabilistic interaction among adjacent variables. Each clique in UGs is defined as a potential function, also regarded as a factor. Moreover, the joint probability distribution is the product of these factors [154]. For a few systems in high-speed trains, interactions among some special variables are non-directional. Thus, UGs can better describe these probability models of faults [149].

In general, UGs are composed of the network structure graph *H* and a factor set Ψ. Nodes in *H* represent random variables describing faults. If any two nodes in a subgraph of *H* are connected by edges, the subgraph is called as a complete subgraph. A factor set in UGs, also named as the potential function $\Psi =\left\{\phi_{1}\left(D_{1}\right),\ldots ,\phi_{K}\left(D_{K}\right)\right\}$, can show interactions among variables. Furthermore, how to improve the fault detectability of UGs is discussed in Reference [155] to prepare for FD tasks of high-speed trains.

Different from other graphs, UGs for the FD applications of high-speed trains are necessary to combine other approaches, such as k-nearest neighbor algorithms, ARs, and so on. Reference [156] proposes the FD scheme based on UGs, by introducing periodograms which are extracted from bearing signals into k-nearest neighbor classifiers, to perform FD tasks of rolling bearings in high-speed trains. It is a new direction to combine UGs with other IFD techniques, and the advantages of UGs in FD fields need to be explored.

#### 3.2.3. Clain Graph

Clain graph (CG) approaches are the natural extension of UGs and DAGs. In comparison with UGs and DAGs, CGs have a stronger ability to express Markov properties [157]. The form of CGs is very special; when certain conditions are satisfied, CGs can be approximated as DAGs or UGs.

According to mathematical expressions of UGs and DAGs, CG can be defined as G=(V,E), and there are no bidirectional edges and no directed cycles in *G*. To be specific, if there are only undirected edges in *G*, CGs will become UGs; if there are only directed edges in *G*, CGs will become DAGs. Suppose that the vertices in *G* can be divided into the ordered sequence $V=B_{1}\cup \cdots \cup B_{k}$, and edges in CGs require to be satisfied:

(1) If $\left\{X,Y\right\}$ is an edge in *G*, and $X,Y\in B_{i}$, then the form of an edge will be X−Y.
(2) If $\left\{X,Y\right\}$ is an edge in *G*, and $X\in B_{i},Y\in B_{j},i<j$, then the form of an edge will be X→Y.

Here, $B_{k}$ represents a block for i=1,…,k. When all blocks $B_{1},B_{2},\ldots ,B_{k}\;\left(k\geq 1\right)$ are connected together, dependency chains can be formed through this connection. In the actual FD procedure, vertex sets in CGs follow the principle of partially ordered sets. Let $\left\{X,V_{1},\ldots ,V_{n},Y\right\}$ be the vertex of CGs, and the subgraph $G_{A}$ can be described:

(8) $$\begin{array}{c} X\to V_{1}-V_{2}-\cdots -V_{n-1}-V_{n}\leftarrow Y, \end{array}$$

in which the subgraph $G_{A}$ is a composite structure in the *G*. Both *X* and *Y* are parent nodes of composite structures, a set $\left\{V_{1},\ldots ,V_{n}\right\}$ is the domain of composite structures, and *n* is the degree of the composite structure.

Based on the above description of CG structures, CGs can be used to simulate the coupling structure of systems in high-speed trains. More specifically, it is easy to simulate the monitoring of multiple subsystems of trains in the form of CGs. In particular, a variable $X_{i}$ is defined as the fault characteristic. In addition, another set of variables $Y_{1},\ldots ,Y_{n}$ is defined as a condition variable that can directly affect $X_{i}$. Besides, variables $Z_{1},\ldots ,Z_{t}$ are defined to describe FD procedures, which have no direct influence on the variable $X_{i}$. Let G=(V,E) as a CG to describe the system structure. Note that it is the one-to-one relationship between the set of nodes and $\left\{X_{i}\right\}\cup \left\{Y_{1},\ldots ,Y_{n}\right\}\cup \left\{Z_{1},\ldots ,Z_{t}\right\}$.

In addition, the probability distribution in CGs should include the probabilities of variables (i.e., $x_{i},y_{1},\ldots ,y_{n}$), and the conditional probability used to judge whether there is a component failure is

(9) $$\begin{array}{c} P(X_{i}\leq x_{i}|Y_{1}=y_{1},\ldots ,Y_{n}=y_{n})=p. \end{array}$$

When *p* is close to 0, the component monitored by $x_{i}$ may be abnormal. When *p* is close to 1, the component is normal.

Reference [158] proves the Markov property of CGs, and even the conditional independence of strict integer probability distribution, is expressed by CGs. Evidently, this work promotes the development of CGs. To explain the causality of CG structures, Reference [159] proves the decomposition of joint probability distributions of CGs. Because CG structures are also relatively complex, Reference [160] developed a structure learning approach to training structures and parameters of CGs. Naturally, CGs are suitable for describing behaviors of complex systems. For example, a CG framework was developed by Reference [161] to detect faults in electrical systems.

The direct separation criterion of global Markov property in CGs has always been the attention of academics because it is easily applied through the simple separation standard in practice. Therefore, research on related separation standards can extend the application of CGs in high-speed trains.

#### 3.2.4. Fault Tree Analysis

In addition to the above graphs, several other graphs thrived during the 1960s. Among these techniques, with a mixture of qualitative analysis and a specific logic diagram, fault tree analysis (FTA) approaches are causal models based on qualitative analysis [35]. Different from CGs, FTA methods can conduct reasoning analysis step-by-step when high-speed trains fail and then identify fault causes [147]. In addition, FTA techniques mainly discuss two kinds of faults: (1) the fault is not recovered by itself; and (2) the existence of the abnormal state, in which faults in high-speed trains are caused by external conditions. The fault automatically disappears when the abnormal conditions are restored.

Two core tasks on FTA modeling are: (1) to determine the top event; and (2) to find the boundary condition. The top event in FTA indicates FD events in high-speed trains, and the selection of top events is particularly important for FTA modeling [162]. Besides, boundary conditions include system boundaries, initial conditions, disallowed events, and existing assumptions. These conditions could mainly reflect the current detailed information of systems, to judge whether the system is in a normal state [135].

The FD implementation of FTA begins from the top FD event, through the way from top to bottom, gradually delivering to the bottom of FD events. Through reasoning to find out the cause and influences of each level of events, FTA approaches can further determine the cause of top-level FD events. Several principles using FTA are given as follows:

(1) Fault types ought to be as extensive as possible.
(2) The analysis of fault events should be as detailed as possible.
(3) The principle of layer by layer transmission should be followed (please pay attention to the function of the gates in FTA methods).
(4) Only gates in FTA approaches can be connected to events.

The function of “AND” gates is that, if all the input events occur, the output event will occur. On the contrary, the function of “OR” gates is that, if any input event occurs, the output event will occur. There are at least two input events when using “OR” gates or “AND” gates [163]. Suppose *n*-th input event exists in FTA frameworks, and the probability of using “AND” gates is

(10) $$\begin{array}{c} V=U_{1}+U_{2}+\cdots +U_{n}, \end{array}$$

(11) $$\begin{array}{cc} P(V) & =P(U)+P(A)-P(U\cap A)\\ \; & =P(U)+P(A)+P(U)P(A|U). \end{array}$$

Similarly, the probability of using “OR” gates is

(12) $$\begin{array}{c} V=U_{1}\times U_{2}\times \cdots \times U_{n}, \end{array}$$

(13) $$\begin{array}{c} P(V)=P(U)P(A|U)=P(A)P(U|A). \end{array}$$

Since static FTA techniques are difficult to satisfy the real-time requirement in high-speed trains, Reference [164] extends FTA into the dynamic FD scheme by incorporating the additional gates to diagnose faults in trains. Generally speaking, standard FTA techniques cannot express time-related behaviors. On this account, the work in Reference [165] proposes a time-dependent FTA technique, by using timed statecharts to describe the time-variant system, to solve the optimal control problem of the high-speed railway crossing. On this basis, an extended FTA combined with Petri nets is developed in Reference [166], simultaneously considering time parameters, to address FD issues of train dynamic systems with repairable multi-state components. Recent work has also reported on techniques that integrate other approaches into the standard FTA, e.g., Reference [167] designed an IFD scheme based on FTA, mainly using fuzzy set theory to overcome the negative influence from inaccurate expert experience, to detect faults in the Chinese Train Control System Level 3 (CTCS-3).

### 3.3. Fuzzy Theory in Qualitative IFD Approach of High-Speed Trains

In contrast to the above methods requiring explicit fault information, IFD approaches based on fuzzy theory are able to handle some extremely inaccurate diagnostic knowledge of high-speed trains. Because of a complicated relationship among fault modes and system mechanisms in trains, the boundaries among failures become blurred [168]. In recent years, fuzzy theory has been attractive [169,170,171,172] in diagnosing to high-speed trains.

Fuzzy theory is mainly composed of fuzzy set theory, fuzzy logic, fuzzy reasoning, and fuzzy control system. According to the viewpoint in Reference [173], classical cybernetics overemphasize accuracy and can not deal with complex systems. Thus, there is an urgent need for a new theory to describe fuzzy relationships, and fuzzy theory has been established to deal with various challenges of classical cybernetics.

Based on the fuzzy idea, the value of 1 or 0 in the general set is described as a more fuzzy concept, i.e., the arbitrary mapping from any element *u* to a closed interval in the domain *U*. The fuzzy description is more convenient to express fuzzy relationships among phenomenons and causes of faults [174]. In addition, fuzzy logic (FL), also regarded as a fuzzy extension of non-fuzzy multi-valued logic, can extend explicit relationships between “having” and “not having” to fuzzy relationships.

With the continuous improvement of fuzzy theory, its applications in transportation fields are increasing gradually. One of the more successful applications to high-speed trains is the fuzzy logic reasoning system, mainly including pure fuzzy logic systems, “Takagi-Sugeno” fuzzy logic systems, and “Mandani” fuzzy logic systems [175]. There is a unified implementation procedure of these FD systems, i.e.,

- Step 1: Extract the fault characteristic fuzzy vector $X=(\mu_{x1},\ldots ,\mu_{xn})$ and fault cause fuzzy vector $Y=(\mu_{y1},\ldots ,\mu_{ym})$. There are *n* characteristics generated by faults of high-speed trains, in which the domain is $U=(x_{1},\ldots ,x_{n})$. The variable describing the *i*-th characteristic is $u_{i}$, and its membership function is $\mu_{ui}$. On this basis, $X=(\mu_{x1},\ldots ,\mu_{xn})$ is established as the fault characteristic fuzzy vector. Besides, there may be *m* fault causes when faults of systems occur, its domain is $V=(y_{1},\ldots ,y_{m})$. The variable describing the *j*-th cause is $v_{j}$, and its membership function is $\mu_{yj}$. On this basis, $Y=(\mu_{y1},\ldots ,\mu_{ym})$ is established as the fault cause fuzzy vector.
- Step 2: Calculate the diagnostic matrix *R*. There is a connection among many facts in FD procedures, so the relationship among faults and fault symptoms is established through the diagnostic relation matrix *R* from *U* to *V*. In addition, the above relationship can be represented by an ordered pair (x,y), where the Cartesian product set of *U* domain and *V* domain is $U\times V=\left\{(x,y)|x\in X,y\in Y\right\}$. Furthermore, *R* is a fuzzy set defined on U×V, as follows:
  (14) $$R=\left[\begin{array}{cccc} r_{11} & r_{12} & \cdots \; & r_{1n}\\ r_{21} & r_{22} & \cdots \; & r_{2n}\\ \vdots \; & \vdots \; & \ddots \; & \vdots \;\\ r_{m1} & r_{m2} & \cdots \; & r_{mn} \end{array}\right],$$
  where the fuzzy set represents the causal relationship among causes and characteristics of fault.
- Step 3: Implement the fuzzy reasoning. Suppose *R* is a fuzzy relation from *U* to *V*. By means of fuzzy relation R(x,y)=U→V=U×V, the fuzzy set $V^{{}^{\prime }}$ on *V* can be calculated. With the help of characteristic fuzzy vectors $U^{{}^{\prime }}$, the new domain of fault fuzzy vectors can be calculated via:
  (15) $$\begin{array}{c} V^{{}^{\prime }}=U^{{}^{\prime }}\circ \left(U\to V\right)=V^{{}^{\prime }}\circ R, \end{array}$$
  where ∘ is the composition operator of fuzzy relation.
- Step 4: Perform the fuzzy FD. Faults can be matched with their causes by the maximum membership criterion. Specifically, if the element $\mu^{*}$ belongs to *U* domain and satisfies $\mu_{c}\left(\mu^{*}\right)=\max\left[\mu_{c}\left(\mu_{1}\right),\ldots ,\mu_{c}\left(\mu_{n}\right)\right]$, then the fault characteristic $X_{i}$ will be matched with the fault cause $Y_{j}$.

Critical technological advances in fuzzy theory, such as IFD via fuzzy reasoning, promote the applicability of fuzzy theory in high-speed trains. Reference [168] uses fuzzy reasoning to identify faults in the bearing of high-speed trains, providing for engineers with a thorough grasp for the next maintenance decision. In high-speed trains, monitoring devices of choke adapter transformer are outside, and their characteristic signals are difficult to be continuously monitored. One solution given in Reference [170] develops a fuzzy IFD approach based on the combination of rough set theory and fuzzy reasoning to detect faults in the transformer. To test the FD performance of fuzzy theory, an adaptive neuro-fuzzy reasoning system is proposed in Reference [171] to detect several faults in CTCS-3. Research on the adaptive IFD model based on fuzzy theory has attracted attentions, e.g., Reference [172] takes into account many factors, such as uncertain force and resistance disturbance in high-speed trains, and further proposes fuzzy adaptive reasoning to diagnose actuator faults.

For high-speed trains with large-time delay, time-varying, and nonlinear characteristics, qualitative IFD techniques based on fuzzy theory are the promising FD schemes, reflecting in the handling of uncertainty and imprecision aspects. Naturally, combining fuzzy theory with other methods, such as ESs and graphs, can achieve better FD performance. In addition, it is vital to conduct detailed fault cause analysis on the mixed techniques. To an end, Table 2 shows the advantages and limitations of the above techniques to promote their applications in high-speed trains.

## 4. Challenges and Future Trends

Over the past three decades, qualitative IFD approaches have been successfully applied in transportation areas of FD. Currently, qualitative techniques have gotten onto the intelligent stage. However, some challenges need to be addressed, especially in FD of high-speed trains, are given as follows:

- Qualitative IFD techniques are useful for a specific system.
- In qualitative IFD techniques, it is difficult to ensure that all rules are applicable.
- The lower quality of knowledge results in worse FD performance in qualitative IFD techniques.
- With the complexity of system mechanism, knowledge becomes difficult to be extracted and stored.
- Qualitative IFD techniques are difficult to diagnose and detect incipient faults in high-speed trains.
- The diagnostic KB with complete fault knowledge, viewed as a prerequisite for using qualitative IFD techniques, is difficult to be constructed.

In addition to summarizing their limitations and challenges, future research trends centered on qualitative IFD techniques are exposed. One of the recent trends is that qualitative IFD techniques combine other approaches to improve the FD performance. The core of the research is still fault identification, location, and prediction. For another, remote and real-time requirements should be considered in FD procedures of high-speed trains. Here, several advanced trends are listed below.

(1) Management and maintenance of explicit diagnostic knowledge. It is well known that the construction of diagnostic KB about high-speed trains is a huge task. One of the difficulties lies in the need to expose invisible knowledge because most researchers or engineers only use existing technologies and explicit diagnostic knowledge to build a KB containing enough rich information. But, it is not even close to sufficient. Explicit knowledge usually contains the two types of known information discussed in Section 2.2. But, invisible knowledge, especially in the human brain of train maintenance engineers, is also an indispensable knowledge resource. At the moment, there have been many studies aiming at explicit knowledge extraction, but few reports consider invisible knowledge extraction. To overcome this difficulty, it is helpful to construct a complete diagnostic KB from the perspective of knowledge extraction.
(2) Improvements in the quality of known quantitative information in high-speed trains. Based on the analysis in Section 2, a large amount of historical data is recorded during the operation of high-speed trains and then can be converted into fault knowledge through data mining methods. However, these data collected from the onboard information system in high-speed trains often suffer from missing data points. When extracting knowledge from missing data and building a diagnostic KB, it is easy to lose important knowledge. Thus, the selection of appropriate preprocessing techniques can improve the quality of knowledge discovery and monitoring data (will be used in data mining methods to extract fault knowledge), thereby improving the quality of the diagnostic KB and the result provided via qualitative IFD techniques.
(3) Deep knowledge mining, extraction, and application. Shallow knowledge could be summarized from the massive historical data collected from high-speed trains. On the contrary, deep knowledge is helpful to explore relationships among subsystems in high-speed trains, providing the new solution for system level faults. It is expected that qualitative IFD techniques combining deep and shallow knowledge will be further developed in the future, so as to break the constraints of traditional qualitative methods for special applications in system level or component level FD.
(4) The fusion of qualitative IFD and health management approaches. Under some special conditions (e.g., complete diagnostic KB, the transparent and interpretable FD procedure), qualitative IFD approaches can show accuracy results. These conditions are also necessary for health management techniques. One emerging solution for qualitative IFD approaches is to integrate into health management techniques, and the whole framework can be regarded as an autonomous and accurate comprehensive evaluation system for high-speed trains. With the critical advantages of health management, engineers can easily report the dynamic degradation of high-speed trains, providing effective suggestions for train maintenance. However, there are some challenges with the above technology, like system integration, sensor selection and optimal layout, and measurement data fusion. Fortunately, solutions to these challenges can improve the reliability of high-speed trains and reduce the operation cost of systems.
(5) The research and application of integrated qualitative IFD techniques. Some critical systems in high-speed trains usually have complex nonlinear features, such as strong coupling and time-varying parameters. In addition, process uncertainties and external interferences also have negative effects on FD procedures. Therefore, different qualitative IFD approaches need to be integrated to improve the FD effect. However, there are still many problems to be further studied, such as combination principles of different methods, the fuzzy knowledge expression after fusion, etc.
(6) The research and application of distributed qualitative IFD techniques. With the development of materials and technologies, high-speed trains are becoming systematic, continuous, and automated, many distributed frameworks, like distributed open-scale FD systems, are applied in FD procedures of trains. Distributed techniques provide a potential way for large-scale IFD. Through the description, decomposition and allocation of FD tasks, distributed qualitative IFD techniques can be designed for the decentralized and problem-oriented subsystems to overcome challenges in a parallel collaboration. Furthermore, FD schemes based on the fusion of multi-agent techniques and qualitative IFD techniques are also the advanced research topics in FD domains.
(7) The research and application of remote cooperative qualitative IFD techniques. The premise is to integrate computer networks into qualitative IFD techniques, in which multicenter computers as servers work together. With the aid of computer remote monitoring, information transmission, remote IFD techniques are easy to realize the processing, transmission, storage, query, and display of monitoring information in high-speed trains. The successful implementation of remote cooperative qualitative IFD techniques will be helpful for online IFD in high-speed trains, providing real-time results for engineers in the operation center. Based on these results, engineers and experts can adjust maintenance plans of trains. The key to this technique includes remote signal analysis, remote transmission of real-time data, and open ES design.

## 5. Conclusions

This paper has discussed the development of qualitative IFD techniques for high-speed trains and reviewed the basic ideas and several important schemes of qualitative IFD techniques, as well as introduced some recent results in the IFD field of high-speed trains. One major focus has been on the background overview of high-speed trains, including the composition of the core subsystems, system structure, and so on, which are helpful to understand and extract fault knowledge, as well as construct a desired diagnostic KB. Knowledge acquisition from available information in high-speed trains is a difficult task; for this purpose, this survey provides some useful schemes from data mining (used for known quantitative information) and manual extraction (used for known qualitative information) aspects.

Another major focus has been on the qualitative IFD schemes, which are, thanks to their interpretability and good logic performance, more suitable for FD applications to high-speed trains. Several qualitative IFD technique related issues have been identified for further studies, including the modeling issues for diagnostic KB, the learning issues for missing points in known quantitative information, deep knowledge extraction, application issues for the distributed qualitative IFD techniques, implementation issues for remote cooperative qualitative IFD techniques, etc. With the development of qualitative IFD and other emerging techniques, we believe qualitative approaches will continue to be attractive and powerful in FD procedures of high-speed trains.

## Figures and Tables

**Table 1:** All reviews in the field of diagnosis from 1990 to present according to the theme and application of each research.

Method Classification	Reference	Application Scenarios
Quantitative technique [35]		
Model-based	Cheng [36], Zeng [37], Xu [38], Yang [39], Xue [40], Lei [41], Ma [42], Dai [43], Shui [44], Wang [45], Mellit [46], Liu [47]	Permanent magnet machine [36], electronic systems [39,40,48], planetary gearbox [41], nuclear power plants [42], power generation equipment [49], dynamic systems [50], aerospace systems [51], wind turbine [52]
Data driven	Chen [1], Ma [42], Du [49], Huang [53], Yang [54], Henao [55], Niu [56], Norazwan [57], Dai [58]	Traction system [1], nuclear power plants [42], power generation equipment [49], converter [53], aeroengine [54], rotating electrical machines [55], multi-axle speed sensors [56], chemical process systems [57], industrial automation [58]
Signal-based	Chen [36], Zeng [37], Xu [38], Yang [39], Xue [40], Lei [41], Ma [42], Dai [43], Shui [44], Wang [45], Mellit [46], Liu [47]	Permanent magnet machine [36], wind turbines [37,38], electric systems [39,40], planetary gearbox [41], nuclear power plants [42], control systems [44], hydraulic systems [43], rotating machines [45], photovoltalic systems [46], squirrel-cage induction motors [47]
Neural Network	Liu [14,59], Yan [60], Wang [61,62], Wen [63], Amiruddin [64], Fenton [48], Hu [65], Li [66,67], Chen [68], Xie [69]	Rotating machinery [14,59], servo system [60], turbine [63], engineering-related systems [64], electric systems [48], rolling bearing [65], diesel engine [66], motor engine [68], gas turbine [69], running gear system [67]
Machine Learning	Liu [14], Zhang [61], Duan [70], Saufi [62], Hu [66], Li [67]	Rolling bearing [14,65,71], high-speed railway [67]
Artificial Intelligence	Mellit [46], Liu [47], Nandi [72], Wen [63]	Photovoltalic systems [46], squirrel-cage induction motors [47], electrical motors [72], turbine [63]
Petri Nets	Zaytoon [73], Niu [56]	Discrete event systems [73], multi-axle speed sensors [56]
Qualitative technique [35]		
Expert System	Yan [60], Wang [61], Li [66], Chen [68], Xie [69], Lin [59]	Servo system [60], motor engine [68], gas turbine [69], rotating machinery [59], high-speed railway [67]
Fuzzy Theory	Wang [61,74], Li [66], Chen [68], Xie [69], Lin [59]	Diesel engine [66], motor engine [68], rotating machinery [59]
Knowledge-based	Cheng [36], Yang [39], Xue [40], Du [49], Wang [75]	Permanent magnet machine [36], electric systems [39,40,75], power generation equipment [49]
Rule and Case-based	Wang [47], Fenton [48]	Electrical systems [48]
Fault Tree	Wang [61], Zaytoon [73], Chen [68]	Discrete event systems [73], motor engine [68]

**Table 2:** List of advantages and limitations of qualitative IFD techniques.

Method	Advantage	Limitation
RBR	1. Easy-to-understand forms of reasoning 2. Expression of uncertain knowledge 3. Easy interpretation	1. Hard to grasp the overall structure of knowledge 2. Unclear relationships among the rules 3. Lack of flexibility in reasoning
CBR	1. The extended coverage of case bases with the continuous use of systems 2. No rule extraction	1. A complicated knowledge extraction process 2. The low retrieval efficiency in large case base 3. Consistency test of difficult case correction
ES	1. Transparent and interpretable FD procedures 2. No requirements of mathematical equations 3. Easy to determine the fault cause	1. Lack of the self-learning and self-adaptive ability 2. The slow reasoning speed and low efficiency 3. Not ideal for the real-time performance
DG	1. Processing of various uncertainties via graphs 2. More forms of knowledge expressions 3. No need for other reasoning methods	1. Easy to lose vital variables in fault propagations 2. Dependent on the experience and simulation 3. Further simplification of the model
UG	1. The expression from global graphic forms	1. Difficult to accurately locate faults
CG	1. No requirements of mathematical equations 2. Simple and easy to be operated	1. Complex search procedure 2. Easy to lose information
FTA	1. Good logic performance 2. Easy modeling	1. High requirements for failure mechanism 2. Weak real-time processing ability
Fuzzy Theory	1. More solutions with different priorities 2. The ability to analyze uncertainty issues	1. With subjective factors 2. No self-learning ability

## Data Availability

Not Available.

## References

[1] Chen H.T., Jiang B. (2020). A review of fault detection and diagnosis for the traction system in high-Speed trains. IEEE Trans. Intell. Transp. Syst..

[2] Yang C.H., Yang C., Peng T., Yang X.Y., Gui W.H. (2017). A fault-injection strategy for traction drive control systems. IEEE Trans. Ind. Electron..

[3] Tu D.Y., Zheng J.D., Jiang Z.W., Pan H.Y. (2018). Multiscale distribution entropy and t-distributed stochastic neighbor embedding-based fault diagnosis of rolling bearings. Entropy.

[4] Chen Z.W., Ding S.X., Peng T., Yang C.H., Gui W.H. (2018). Fault detection for non-gaussian processes using generalized canonical correlation analysis and randomized algorithms. IEEE Trans. Ind. Electron..

[5] Chen H.T., Jiang B., Lu N.Y. (2019). A newly robust fault detection and diagnosis method for high-speed trains. IEEE Trans. Intell. Transp. Syst..

[6] Guo L., Lei Y.G., Xing S.B., Yan T., Li N.P. (2019). Deep convolutional transfer learning network: A new method for intelligent fault diagnosis of machines with unlabeled data. IEEE Trans. Ind. Electron..

[7] Cheng C., Wang W.J., Luo H., Zhang B.C., Cheng G.L., Teng W.X. (2020). State-degradation-oriented fault diagnosis for high-speed train running gears system. Sensors.

[8] Chen H.T., Jiang B., Zhang T.Y., Lu N.Y. (2020). Data-driven and deep learning-based detection and diagnosis of incipient faults with application to electrical traction systems. Neurocomputing.

[9] Chen H.T., Jiang B., Ding S.X., Lu N.Y., Chen W. (2019). Probability-relevant incipient fault detection and diagnosis methodology with applications to electric drive systems. IEEE Trans. Control Syst. Technol..

[10] Cheng C., Wang J.H., Fu C.X., Zhang B.C. A status assessment model for dynamic system based on cloud evidence reasoning. Proceedings of the IECON 2019.

[11] Chen H.T., Jiang B., Lu N.Y., Chen W. (2018). Real-time incipient fault detection for electrical traction systems of CRH2. Neurocomputing.

[12] Chen H.T., Jiang B., Chen W., Li Z.H. (2020). Edge computing-aided framework of fault detection for traction control systems in high-speed trains. IEEE Trans. Veh. Technol..

[13] Lei Y.G., Lin J., He Z.J., Zuo M.J. (2013). A review on empirical mode decomposition in fault diagnosis of rotating machinery. Mech. Syst. Signal Process.

[14] Liu R., Yang B., Zio E., Chen X.F. (2018). Artificial intelligence for fault diagnosis of rotating machinery: A review. Mech. Syst. Signal Process.

[15] Liu Z.G., Liu K., Zhong J.P., Han Z.W., Zhang W.X. (2020). A high-precision positioning approach for catenary support components with multi-scale difference. IEEE Trans. Instrum. Meas..

[16] Wang X.F., Yang G.H. Event-triggered fault detection observer design for T-S fuzzy systems. IEEE Trans. Fuzzy Syst..

[17] Chen H., Chai Z., Jiang B., Huang B. Data-driven fault detection for dynamic systems with performance degradation: A unified transfer learning framework. IEEE Trans. Instrum. Meas..

[18] Chen H.T., Jiang B., Chen W., Yi H. (2019). Data-driven detection and diagnosis of incipient faults in electrical drives of high-speed trains. IEEE Trans. Ind. Electron..

[19] Shang C., Yang F., Gao X., Huang X., Suykens J.A.K., Huang D. (2015). Concurrent monitoring of operating condition deviations and process dynamics anomalies with slow feature analysis. AIChE J..

[20] Chen H.T., Jiang B., Lu N.Y., Mao Z.H. (2018). Deep PCA based real-time incipient fault detection and diagnosis methodology for electrical drive in high-speed trains. IEEE Trans. Veh. Technol..

[21] Cheng C., Wang J.H., Zhou Z.J., Teng W.X., Sun Z.B., Zhang B.C. A BRB-based effective fault diagnosis model for high-speed trains running gear systems. IEEE Trans. Intell. Transp. Syst..

[22] Zhou D.H., Zhao Y.H., Wang Z.D., He X., Gao M. (2020). Review on diagnosis techniques for intermittent faults in dynamic systems. IEEE Trans. Ind. Electron..

[23] Song L.L. (2016). Synthetically Intelligent Diagnosis Approach of High-Speed Trains Based on the Non-Canonical Knowledge Processing. Ph.D. Thesis.

[24] Cheng C., Qiao X.Y., Luo H., Teng W.X., Gao M.L., Zhang B.C., Yin X.J. (2019). A semi-quantitative information based fault diagnosis method for the running gears system of high-speed trains. IEEE Access.

[25] Lu J.G., Zhang H., Tang X.H. (2019). A novel method for intelligent single fault detection of bearings using SAE and improved D-S evidence theory. Entropy.

[26] Glowacz A., Glowacz W., Kozik J., Piech K., Gutten M., Caesarendra W., Liu H., Brumercik F., Irfan K., Khan Z. (2019). Detection of deterioration of three-phase induction motor using vibration signals, measurement science review. Meas. Sci. Rev..

[27] Delpha C., Diallo D., Samrout A.H., Moubayed N. (2018). Multiple incipient fault diagnosis in three-phase electrical systems using multivariate statistical signal processing. Eng. Appl. Artif. Intell..

[28] Yuan X.F., Li L., Shardt Y., Wang Y.L., Yang C.H. Deep learning with spatiotemporal attention-based LSTM for industrial soft sensor model development. IEEE Trans. Ind. Electron..

[29] Tian E.G., Wang X.M., Peng C. Probabilistic-constrained distributed filtering for a class of nonlinear stochastic systems subject to periodic DOS attacks. IEEE Trans. Circuit. Syst.–I.

[30] Yu W.K., Zhao C.H., Huang B. Stationary subspace analysis based hierarchical model for batch processes monitoring. IEEE Trans. Control Syst. Technol..

[31] Tian E.G., Peng C. Memory-based event-triggering H-infinity load frequency control for power systems under deception attacks. IEEE Trans. Cybernetics.

[32] Delpha C., Chen H., Diallo D. SVM based diagnosis of inverter fed induction machine drive: A new challenge. Proceedings of the 38th Annual Conference on IEEE Industrial Electronics Society.

[33] Wan S.T., Peng B. (2019). An integrated approach based on swarm decomposition, morphology envelope dispersion entropy, and random forest for multi-fault recognition of rolling bearing. Entropy.

[34] Frank P.M. (1990). Fault diagnosis in dynamic systems using analytical and knowledge-based redundancy: A survey and some new results. Automatica.

[35] Zhou D.H., Hu Y.Y. (2009). Fault diagnosis techniques for dynamic systems. Acta Autom. Sinica.

[36] Cheng M., Hang J., Zhang J.Z. (2015). Overview of fault diagnosis theory and method for permanent magnet machine. Chin. J. Electr. Eng..

[37] Zeng J., Chen Y.F., Yang P., Guo H.X. (2018). Review of fault diagnosis methods of large-scale wind turbines. Power Syst. Technol..

[38] Xu J.L., Ge Z.S., Chen Q. (2018). Summary fault diagnosis of wind turbines. Sci. Technol. Vision.

[39] Yang S. (2019). Application and prospect on electrical servo fault diagnosis technology in China. Henan Sci. Technol..

[40] Xue G.H., Wu M. (2010). Research status and development trend of fault diagnosis methods for electromechanical equipment. Coal Eng..

[41] Lei Y.G., Lin J., Zuo M.J., He Z.J. (2014). Condition monitoring and fault diagnosis of planetary gearboxes: A review. Measurement.

[42] Ma J.P., Jiang J. (2011). Applications of fault detection and diagnosis methods in nuclear power plants: A review. Prog. Nucl. Energy.

[43] Dai J.Y., Tang J., Huang S.Z., Wang Y.Y. (2019). Signal-based intelligent hydraulic fault diagnosis methods: Review and prospects. Chin. J. Mech. Eng..

[44] Shui A., Chen W.M., Zhang P., Hu S.R., Huang X.W. Review of fault diagnosis in control systems. Proceedings of the CCDC 2009.

[45] Wang Y.X., Xiang J.W., Markert R., Liang M. (2016). Spectral kurtosis for fault detection, diagnosis and prognostics of rotating machines: A review with applications. Mech. Syst. Signal Process.

[46] Mellit A., Tina G.M., Kalogirou S.A. (2018). Fault detection and diagnosis methods for photovoltaic systems: A review. Renew. Sust. Energy Rev..

[47] Liu Y.Q., Bazzi A.M. (2017). A review and comparison of fault detection and diagnosis methods for squirrel-cage induction motors: State of the art. MISA Trans..

[48] Fenton W.G., McGinnity T.M., Maguire L.P. (2001). Fault diagnosis of electronic systems using intelligent techniques: A review. IEEE Trans. Syst. Man Cybern. C Appl. Rev..

[49] Du J.Q., Zhao M., Yin J., Gu W. (2018). Review of fault diagnosis methods for power plant. Yunnan Electr. Power.

[50] Patton R.J., Chen J. A review of parity space approaches to fault diagnosis. Proceedings of the IFAC SAFEPROCESS Symptoms.

[51] Patton R.J., Chen J. (1994). Review of parity space approaches to fault diagnosis for aerospace systems. Journal Guid. Control Dynam..

[52] Lu B., Li Y.Y., Wu X., Yang Z.Z. A review of recent advances in wind turbine condition monitoring and fault diagnosis. Proceedings of the PEMWA 2009.

[53] Huang L.M., Zhang Q. (2019). Review on fault diagnosis of converter based on data driven. Electr. Technol..

[54] Yang H.F., Jia X.L., Ren S.W. (2016). Review of data-driven aeroengine fault diagnosis and prognosis methods. Aviat. Precis. Manuf. Technol..

[55] Henao H., Cabanas M., Filippetti F., Bruzzese C., Strangas E., Pusca R., Estima J., Riera-Guasp M., Kia S.H. (2014). Trends in fault diagnosis for electrical machines: A review of diagnostic techniques. IEEE Ind. Electron. Mag..

[56] Niu G., Xiong L.J., Qin X.X., Pecht M. (2019). Fault detection isolation and diagnosis of multi-axle speed sensors for high-speed trains. Mech. Syst. Signal Process.

[57] Nor N.M., Hassan C.R.C., Hussain M.A. (2020). A review of data-driven fault detection and diagnosis methods: Applications in chemical process systems. Rev. Chem. Eng..

[58] Dai X.W., Gao Z.W. (2013). From model, signal to knowledge: A data-driven perspective of fault detection and diagnosis. IEEE Trans. Ind. Inform..

[59] Liu Y.J., Wang Z.J. (2010). Overview of methods for rotating machinery fault diagnosis. J. Suzhou Vocat. Univ..

[60] Yan Z.Q. (2005). Intelligent fault diagnosis and Simulation of servo system. Electromech. Eng. Technol..

[61] Wang F.T., Ma X.J., Zou Y.K. (2003). An overview on intelligent technique of fault diagnosis. Mach. Tools Hydraul..

[62] Saufi S.R., Ahmad Z.A.B., Leong M.S., Lim M.H. An intelligent bearing fault diagnosis system: A review. Proceedings of the Engineering Application of Artificial Intelligence Conference 2018.

[63] Jing Y.W., Li L.F. (2014). Research overview on intelligent fault diagnosis technology of turbine. Appl. Mech. Mater..

[64] Amiruddin A.A.A.M., Zabiri H., Taqvi S.A.A., Tufa L.D. (2018). Neural network applications in fault diagnosis and detection: An overview of implementations in engineering-related systems. Neural Comput. Appl..

[65] Hu D.Q. (2019). Summary of rolling bearing fault diagnosis methods. Internal Combust. Engine Parts.

[66] Li B.Y., Jiang G.H., Chen B.W., Wang P.F. (2019). Overview of diesel engine fault diagnosis technology. Ship Mater. Market.

[67] Li X.M., He D.Q., Deng J.X., Miao J. (2015). Summary of fault diagnosis methods for bogies of urban rail transit vehicles. Eq. Manuf. Technol..

[68] Chen L.X., Liu F.J., He Y.L. (2010). An overview on the intelligent fault diagnosis research of automobile engineering. Manuf. Automat..

[69] Xie C.L., Dai J.M. (2010). The research overview and prospects of gas turbine fault diagnosis technique. Turb. Technol..

[70] Duan L.X., Xie M.Y., Wang J.J., Bai T.B. (2018). A Deep learning enabled intelligent fault diagnosis: Overview and applications. J. Intell. Fuzzy Syst..

[71] Zhang X.Y., Luan Z.Q., Liu X.L. (2017). Review on fault diagnosis of rolling bearing based on deep learning. Eq. Manage. Maintenance.

[72] Nandi S., Toliyat H.A., Li X.D. (2005). Condition monitoring and fault diagnosis of electrical motor-A review. IEEE Trans. Energy Conver..

[73] Zaytoon J., Lafortune S. (2013). Overview of fault diagnosis methods for Discrete Event Systems. Annu. Rev. Control.

[74] Wang C.J., Sun G.Z. (1998). A review of intelligent models for fault diagnosis. Water Conservancy Electr. Power Mach. Water Conservancy Electr. Power Mach..

[75] Wang R.C. (2019). Survey of knowledge based fault diagnosis methods for power systems. Sci. Technol. Innov..

[76] Liu Z.L., Wang H., Liu J.J., Qin Y., Peng D.D. Multi-task learning based on lightweight 1DCNN for fault diagnosis of wheelset bearings. IEEE Trans. Instrum. Meas..

[77] Harmouche J., Delpha C., Diallo D. (2014). Incipient fault detection and diagnosis based on Kullback-Leibler divergence using principal component analysis: Part I. Signal Process..

[78] Chen J.W., Liu Z.G., Wang H.R., Núñez A., Han Z.W. (2018). Automatic defect detection of fasteners on the catenary support device using deep convolutional neural network. IEEE Trans. Instrum. Meas..

[79] Duan A., Guo L., Gao H., Wu X., Dong X. Deep Focus Parallel Convolutional Neural Network for Imbalanced Classification of Machinery Fault Diagnostics. IEEE Trans. Instrum. Meas..

[80] Cheng C., Qiao X.Y., Teng W.X., Gao M.L., Luo H. (2020). Principal component analysis and belief-rule-base aided health monitoring method for running gears of high-speed train. Sci. China Inform. Sci..

[81] Chen H.T., Jiang B., Ding S.X. A broad learning aided data-driven framework of fast fault diagnosis for high-speed trains. IEEE Intell. Transp. Syst. Magazine.

[82] Zhao H.Y., Liang J.Y., Liu C.Q. (2020). High-speed EMUs: Characteristics of technological development and trends. Engineering.

[83] Wang J.F., Wu H.F., Yang T.Y., Zhang L., Xing Y. (2019). Bidirectional three-phase DC-AC converter with embedded DC-DC converter and carrier-based PWM strategy for wide voltage range applications. IEEE Trans. Ind. Electron..

[84] Cheng C., Liu M., Zhang B.C., Yin X.J., Fu C.X., Teng W.X. Health assessment of high-speed train running gear system under complex working conditions based on data-driven model. Math. Prob. Eng..

[85] Cheng C., Wang J.H., Teng W.X., Gao M.L., Zhang B.C., Yin X.J., Luo H. (2018). Health status prediction based on belief rule base for high-speed train running gear system. IEEE Access.

[86] Chen H.T., Jiang B., Lu N.Y., Chen W. (2020). Data-Driven Detection and Diagnosis of Faults in Traction Systems of High-Speed Trains.

[87] Yang F., Habibullah M.S., Zhang T., Xu Z., Lim P., Nadarajan S. (2016). Health index-based prognostics for remaining useful life predictions in electrical machines. IEEE Trans. Ind. Electron..

[88] Delpha C., Diallo D. Incipient fault detection and diagnosis: A hidden information detection problem. Proceedings of the ISIE 2015.

[89] Tai L.G., Guo H.Y., Zhong Y.X., Li D.Q. (2007). Knowledge acquisition method for case-knowledge-based product family design. J. Harbin I Technol..

[90] Caceres S., Henley E.J. (1976). Process failure analysis by block diagrams and fault trees. Ind. Eng. Chem..

[91] Barrientos F., Sainz G. (2012). Interpretable knowledge extraction from emergency call data based on fuzzy unsupervised decision tree. Knowl.-Based Syst..

[92] Baysal M., Gunay M.E., Yildirim R. (2017). Decision tree analysis of past publications on catalytic steam reforming to develop heuristics for high performance: A statistical review. Int. J. Hydrogen Energy.

[93] Thomas M.C., Zhu W., Romagnoli J.A. (2017). Data mining and clustering in chemical process databases for monitoring and knowledge discovery. J. Process Control.

[94] Castro A.R.G. (2004). Knowledge Extraction from Artificial Neural Networks: Application to Transformer Incipient Fault Diagnosis. Ph.D. Thesis.

[95] Shi F., Sun S., Xu J. (2012). Employing rough sets and association rule mining in KANSEI knowledge extraction. Inform. Sci..

[96] Feng L., Li T., Ruan D., Gou S. (2011). A vague-rough set approach for uncertain knowledge acquisition. Knowl.-Based Syst..

[97] Wang H., Kwong S., Jin Y., Wei W., Man K.F. (2005). Multi-objective hierarchical genetic algorithm for interpretable fuzzy rule-based knowledge extraction. Fuzzy Sets Syst..

[98] Tan K.C., Yu Q., Heng C.M., Lee T.H. (2003). Evolutionary computing for knowledge discovery in medical diagnosis. Artif. Intell. Med..

[99] Zhang B., Yang C., Zhu H., Shi P., Gui W. (2018). Controllable-domain-based fuzzy rule extraction for copper removal process control. IEEE Trans. Fuzzy Syst..

[100] Zhu P., Hu Q. (2013). Rule extraction from support vector machines based on consistent region covering reduction. Knowl.-Based Syst..

[101] Cintra M.E., Camargo H.A., Monard M.C. (2016). Genetic generation of fuzzy systems with rule extraction using formal concept analysis. Inform. Sc..

[102] Katipamula S., Brambley M. (2005). Review article: Methods for fault detection, diagnostics, and prognostics for building systems—A review, Part I. HVAC R Res..

[103] Venkatasubramanian V., Rengaswamy R., Kavuri S.N. (2003). A review of process fault detection and diagnosis Part II: Qualitative models and search strategies. Comput. Chem. Eng..

[104] Feng Z.C., Zhou Z.J., Hu C.H., Chang L.L., Hu G.Y., Zhao F.J. (2018). A new belief rule base model with attribute reliability. IEEE Trans. Fuzzy Syst..

[105] Isermann R. Model base fault detection and diagnosis methods. Proceedings of the 1995 American Control Conference.

[106] Ishibuchi H., Nozaki K., Yamamoto N., Tanaka H. (1995). Selecting fuzzy if-then rules for classification problems using genetic algorithms. IEEE Trans. Fuzzy Syst..

[107] Wang W.L., Yang M., Seong P.H. (2016). Development of a rule-based diagnostic platform on an object-oriented expert system shell. Ann. Nucl. Energy.

[108] Chen H.T., Jiang B., Ding S.X., Huang B. Data-driven fault diagnosis for traction systems in high-speed trains: A survey, challenges, and perspectives. IEEE Trans. Intell. Transp. Syst..

[109] Fayyad U., Smyth P. (1993). Image batabase exploration: Progress and challenges. Knowl. Discov. Databases Workshop.

[110] Han J., Pei J., Mortazavi-Asl B., Chen Q., Dayal U., Hsu M.C. Freespan: Frequent pattern-projected sequential pattern mining. Proceedings of the International Conference on Knowledge Discovery and Data Mining (KDD00).

[111] Agrawal R., Imielienski T., Swami A. Mining association rules between sets of items in large databases. Proceedings of the Conf. on Management of Data.

[112] Hu H., Zhang J.W. Research and application on algorithms of data mining for EMU malfunction’s data under cloud computing environment. Proceedings of the New Network Technol. & Appl..

[113] Ren J.H., Liu F., Hu H. Design and implementation of association rules in fault diagnosis of high-speed railway in China. Proceedings of the AMEIT 2017.

[114] Zhao H., Chen H., Dong W., Sun X.Y., Ji Y.D. Fault diagnosis of rail turnout system based on case-based reasoning with compound distance methods. Proceedings of the CCDC 2017.

[115] Yang B.S., Jeong S.K., Oh Y.M., Tan A.C.C. (2004). Case-based reasoning system with Petri nets for induction motor fault diagnosis. Expert Syst. Appl..

[116] Zhong Z.W., Xu T.H., Wang F., Tang T. (2018). Text case-based reasoning framework for fault diagnosis and predication by cloud computing. Math. Probl. Eng..

[117] Yang L.B., Xu T.H., Wang Z.X. Agent based heterogeneous data integration and maintenance decision support for high-speed railway signal system. Proceedings of the ITSC 2014.

[118] Park Y.M., Kim G.W., Sohn J.M. (1997). A logic based expert system for fault diagnosis of power system. IEEE Trans. Power Syst..

[119] Hernandez C., Arjona M.A., Dong S.H. (2008). Object-oriented knowledge-based system for distribution transformer design. IEEE Trans. Magn..

[120] Lee H.J., Ahn B.S., Park Y.M. (2000). A fault diagnosis expert system for distribution substations. IEEE Trans. Power Deliver..

[121] Kumamoto H., Ikenchi K.J., Inoue K., Henley E.J. (1984). Application of expert system techniques to fault diagnosis. The Chem. Eng. J..

[122] Cardozo E., Talukdar S.N. (1988). A distributed expert system for fault diagnosis. IEEE Trans. Power Syst..

[123] Musgrave J.L., Guo T.H., Wong E., Duyar A. (1996). Real-time accommodation of actuator faults on a reusable rocket engine. IEEE Trans. Control Syst. Technol..

[124] Zhou D.X., Xie X.M. (2011). Research of fault diagnosis expert system inference engine based on extension rule. Comput. Meas. Control.

[125] Krishnamurthi M., Phillips D.T. (1992). An expert system framework for machine fault diagnosis. Comput. Ind. Eng..

[126] Bergman S., Astrom K.J. Fault detection in boiling water reactors by noise analysis. Proceedings of the Power Plant Dynamics, Control and Testing Symposium.

[127] Wu J.L., Li H.Z. (1995). Hierachical structure analysis on knowledge representation in expert system. J. Decis. Making Decis. Support Syst..

[128] Yan S.R. (2010). A kind of study program of fault diagnosis expert system for brake of high-speed train. Comput. Knowl. Technol..

[129] Wu J.D., Wang Y.H., Bai M.R. (2007). Development of an expert system for fault diagnosis in scooter engine platform using fuzzy-logic inference. Expert Syst. Appl..

[130] Shendy M.E., Alan S.M. (2000). A fuzzy expert system for fault detection in statistical process control of industrial processes. IEEE Trans. Syst. Man Cybern. Part C.

[131] Song X.D., Shao W., Qiu Z.Z., Chen Y.X. (2013). Study on fuzzy inference method for fault diagnosis expert system. Adv. Mater. Res..

[132] Yang J.B., Liu J., Wang J., Sii H.S., Wang H.W. (2006). Belief rule-base inference methodology using the evidential reasoning approach-RIMER. IEEE Trans. Syst. Man Cybern. Part A.

[133] Xu J.P., Zhong Z.Q., Xu L. (2015). ISHM-oriented adaptive fault diagnostics for avionics based on a distributed intelligent agent system. Int. J. Syst. Sci..

[134] Spiegelhalter J.D., Lauritzen S. (1990). Sequential updating of conditional probabilities on directed graphical structures. Networks.

[135] Pearl J. (1995). Causal diagrams for empirical research. Biometrika.

[136] Lunn J.D., Thomas A., Best N., Spiegelhalter D. (2000). WinBUGS-A Bayesian modelling framework: Concepts, structure, and extensibility. Stat. Comput..

[137] Djeziri M.A., Bouamama B.O., Merzouki R. (2009). Modelling and robust FDI of steam generator using uncertain bond graph model. J. Process Control.

[138] Ghosh N., Bhanu B. (2014). Evolving bayesian graph for three-dimensional vehicle model building from video. IEEE Trans. Intell. Transp. Syst..

[139] Richardson T., Spirtes P. (2002). Ancestral graph Markov models. Ann. Statist..

[140] Liu P.P., Zuo H.F., Su Y., Sun J.Z. (2013). Review of research progresses for graph-based models in fault diagnosis method. Chin. Mech. Eng..

[141] Xie G., Wang X., Xie K.M. SDG-based fault diagnosis and application based on reasoning method of granular computing. Proceedings of the Chinese Control & Decis. Conference.

[142] Zuo J.Y., Chen Z.K. (2014). Sensor configuration and test for fault diagnoses of subway braking system based on signed digraph method. Chin. J. Mech. Eng..

[143] Nam D.S., Han C., Jeong C.W., Yoon E. (1996). Automatic construction of extended symptom fault associations from the signed digraph. Comput. Chem. Eng..

[144] Togari Y., Yonezu S., Yongya A., Hashimoto Y. (1991). Faults diagnosis utilizing a three-layer signed directed graph. Kagaku Kogaku Ronbun..

[145] Bouamama B.O., Biswas G., Loureiro R., Merzouki R. (2014). Graphical methods for diagnosis of dynamic systems: Review. Annu. Rev. Control.

[146] Pearl J. Bayesian networks: A model of self-activated memory for evidential reasoning. Proceedings of the CogSci 1985.

[147] Bobbio A., Portinale L., Minichino M., Ciancamerla E. (2001). Improving the analysis of dependable systems by mapping fault trees into bayesian networks. Reliab. Eng. Syst. Saf..

[148] Richardson T.S., Robins J.M., Wang L.B. (2018). Discussion of “Data-driven confounder selection via Markov and Bayesian network” by Hggstrm. Biometrics.

[149] Pelc A. (1991). Undirected graph models for system-level fault diagnosis. IEEE Trans. Comput..

[150] Zhao J.J., Zheng W. Study of fault diagnosis method based on fuzzy Bayesian network and application in CTCS-3 train control system. Proceedings of the ICIRT2013.

[151] Cai B.P., Liu Y., Xie M. (2016). A dynamic-bayesian-network-based fault diagnosis methodology considering transient and intermittent faults. IEEE Trans. Autom. Sci. Eng..

[152] Wu Y.K., Jiang B., Lu N.Y., Zhou Y. (2015). Bayesian network based fault prognosis via bond graph modeling of high-speed railway traction device. Math. Probl. Eng..

[153] Cheng Y., Xu T.H., Yang L.B. Bayesian network based fault diagnosis and maintenance for high-speed train control systems. Proceedings of the QR2MSE 2013.

[154] Liu J.W., Li H.E., Luo X.L. (2014). Representation theory of probabilistic graphical models. Comput. Sci..

[155] Maheshwari S.N., Hakimi S.L. (1976). On models for diagnosable systems and probabilistic fault diagnosis. IEEE Trans. Comput..

[156] Wang T., Lu G.L., Yan P. Fault diagnosis of rolling bearings based on undirected weighted graph. Proceedings of the PHM-PARIS 2019.

[157] Drton M. (2009). Discrete chain graph models. Bernoulli.

[158] Lauritzen S.L., Wermuth N. (1989). Graphical models for associations between variables, some of which are qualitative and some quantitative. Ann. Statist..

[159] Lauritzen S.L. (2002). Chain graph models and their causal interpretations. J. R. Statist. Soc..

[160] Ma Z., Xie X., Geng Z. (2008). Structural learning of chain graphs via decomposition. J. Mach. Learn. Res..

[161] Flaccadoro D., Cervellera C., Bosia G., Riccomagno E. (2014). Modelling of fault detection and diagnostics for hybrid bus using Chain graph models. Qual. Reliab. Eng. Int..

[162] Song L.L., Wang T.Y., Song X.W., Xu L., Song D.G. (2015). Research and application of FTA and Petri nets in fault diagnosis in the pantograph-type current collector on CRH EMU trains. Math. Probl. Eng..

[163] Ku B.H., Cha J.M. (2011). Reliability assessment of electric railway substation by using minimal cut sets algorithm. J. Int. Council Electr. Eng..

[164] Liu X., Shahidehpour M., Cao Y.J., Li Z.Y., Tian W. (2015). Reliability assessment of electric railway substation by using minimal cut sets algorithm. IEEE Trans. Smart Grid.

[165] Magott J., Skrobanek P. (2012). Timing analysis of safety properties using fault trees with time dependencies and timed state-charts. Reliab. Eng. Syst. Saf..

[166] Khanh Nguyen T.P., Beugin J., Marais J. (2015). Method for evaluating an extended fault tree to analyze the dependability of complex systems: Application to a satellite-based railway system. Reliab. Eng. & Syst. Saf..

[167] Jiang L., Wang X.M., Liu Y.L. (2018). Reliability evaluation of the Chinese train control system level 3 using a fuzzy approach. Proc. I Mech..

[168] Ouyang C.D., Zhang J., Zhao H.M. (2020). The application of fuzzy control system model in bearing fault diagnosis. Eng. Eq. Mater..

[169] Zhang L.L., Wu Y. (2012). An overview of fuzzy theory. Silicon Valley.

[170] Zhang N.Q., Yang S.W., Xu Z.Q., Cui Y. (2017). Fault diagnosis of choke adapter transformer based on rough set and fuzzy inference for high-speed railway. Res. Dev..

[171] Zuo Z.H., Wang K.F., Wei Y.H., Zhao X. Wireless connection timeout fault diagnosis of Chinese train control system using adaptive neuro-fuzzy inference system. Proceedings of the CAC 2017.

[172] Wang M.R., Song Y.D., Song Q., Han P. Fuzzy-adaptive fault-tolerant control of high speed train considering traction/braking faults and nonlinear resistive forces. Proceedings of the ISNN 2011.

[173] Zadeh L.A. (1965). Fuzzy sets. Inform. Control.

[174] Caesarendra W., Pratama M., Kosasih B., Tjahjowidodo T., Glowacz A. (2018). Parsimonious network based on a fuzzy inference system (PANFIS) for time series feature prediction of low speed slew bearing prognosis. Appl. Sci..

[175] Pan J., Desouza G.N. (1998). FuzzyShell: A large-scale expert system shell using fuzzy logic for uncertainty reasoning. IEEE Trans. Fuzzy Syst..

